# Different action of glucocorticoid receptor in adipose tissue remodelling to modulate energy homeostasis by chronic restraint stress

**DOI:** 10.1186/s12944-025-02539-0

**Published:** 2025-03-27

**Authors:** Yinghua Luo, Qinyu Liu, Yaqian Mao, Junping Wen, Gang Chen

**Affiliations:** 1 Shengli Clinical Medical College of Fujian Medical University, Fuzhou, China; 2 Department of Endocrinology, Fujian Provincial Hospital, Fuzhou University Affiliated Provincial Hospital, Fuzhou, China

**Keywords:** Glucocorticoid receptor, Biological stress, Adipose tissue, Thermogenesis

## Abstract

**Background:**

Chronic stress in daily life is a well-known trigger for various health issues. Despite advancements in obesity research, the mechanisms governing lipid metabolism in adipose tissue during cachexia remain poorly understood.

**Methods:**

A chronic restraint stress (CRS) model was used to induce significant physiological and psychological stress in mice. Mice were subjected to 6 h of restraint daily in 50 mL plastic tubes for seven consecutive days. A fasting control group was included for comparison. Post-stress assessments included behavioural tests, glucose and insulin tolerance tests and indirect calorimetry. Blood and adipose tissue samples were collected for mRNA and protein analyses.

**Results:**

CRS induced significant psychological and physiological changes in mice, including depression-like behaviours, weight loss and reduced insulin sensitivity. Notably, CRS caused extensive adipose tissue remodelling. White adipose tissue (WAT) underwent significant ‘browning’ accompanied by an increase in the expression of thermogenic proteins. This counteracted the stress-induced ‘whitening’ of brown adipose tissue (BAT), which exhibited impaired thermogenesis and functionality, thereby maintaining energy balance systematically. The glucocorticoid receptor (GR) plays a crucial role in lipid metabolism regulation during these changes. GR expression levels were inversely correlated in BAT and WAT, but aligned with the expression patterns of thermogenic proteins across adipose tissues. These findings suggest that under chronic metabolic stress, GR mediates tissue-specific responses in adipose tissues, driving functional and phenotypic transitions in BAT and WAT to maintain energy homeostasis.

**Conclusions:**

This study provides novel insights into the contrasting thermogenic phenotypes of BAT and WAT under emaciation and highlights the critical role of GRs in adipose tissue remodelling during CRS and its potential as a therapeutic target. Addressing GR-mediated changes in adipose tissues may help alleviate BAT dysfunction in cachexia and promote WAT browning, enhancing metabolic stress resistance.

**Supplementary Information:**

The online version contains supplementary material available at 10.1186/s12944-025-02539-0.

## Introduction

Modern society’s increasing competition and mounting psychological and physiological pressures have led to the widespread prevalence of chronic stress, characterised by persistent feelings of pressure and being overwhelmed over extended periods [1]. Chronic stress triggers a cascade of cellular, physiological and behavioural effects, resulting in various health issues, including emotional disorders [2, 3], binge eating [4, 5] or anorexia [6, 7], endocrine and metabolic disorders [8, 9], immunologic dissonance [10–12] and even cancer development [13–15].

Concurrently, metabolic abnormalities within different adipose tissue depots frequently result in chronic stress-related obesity or cachexia. Adipose tissue is traditionally classified into two primary types: brown adipose tissue (BAT), responsible for non-shivering thermogenesis and white adipose tissue (WAT), the primary site of energy storage [16, 17]. Moreover, a third type, beige adipocytes, originates from white adipocyte progenitors and can undergo browning in response to specific stimuli, thereby acquiring characteristics of brown adipocytes [18]. Brown adipocytes have an abundance of multichambered lipid droplets and elevated mitochondrial content, indicative of their ability to enhance energy expenditure [19]. This is facilitated by the presence of uncoupling protein 1 (UCP1) [20], enabling the dissipation of the proton gradient across the mitochondrial inner membrane, thereby decoupling respiration from ATP synthesis [21]. Adipose tissue functions not only as an energy reservoir but also as an active endocrine organ, regulating lipid metabolism, thermoregulation and glucose homeostasis [22]. While considerable research has focused on adipose tissue metabolism in obesity, limited attention has been given to its role during emaciation, a state characterised by reduced adipose mass and depot-specific alterations in adipocyte type and function.

Glucocorticoids (GCs), the primary stress hormones particularly in chronic stress, are secreted in response to psychological and physiological stressors and mediate their effects through the glucocorticoid receptors (GRs), a ligand-activated transcription factor. The GC-GR signalling network involves multiple genomic and non-genomic pathways influenced by exposure duration, adipose tissue location and species-specific factors [23]. GR’s transcriptional activity exhibits strong tissue specificity due to its dependence on environmental determinants. The hypothalamic-pituitary-adrenal (HPA) axis play a critical role in chronic stress by regulating GCs secretion through a cascade of hormonal events [24]. GRs influence adipogenesis and lipolysis, but their dual role remains contentious due to the complexity of these processes [25–28].

This study investigates the physiological and metabolic alterations under chronic stress and primarily explores the lipid metabolism and underlying mechanisms, using a chronic restraint stress (CRS) model [29, 30] to induce significant psychological and physiological trauma in mice. Alongside behavioural changes and weight loss, CRS led to pronounced adipose tissue remodelling. Notably, BAT exhibited significant ‘whitening’ and thermogenic dysfunction, while WAT demonstrated adaptive browning. Diverging from previous fat-related research, this study provides novel insights into the contrasting thermogenic phenotypes of BAT and WAT under cachectic conditions and underscores the pivotal role of GR in adipose tissue remodelling.

## Materials and methods

### Mice care

C57BL/6J male and female mice (8 weeks old) were obtained from GemPharmatech (Jiangsu, China) and housed in groups of five in a specific pathogen-free facility. Mice were maintained under a 12-hour light-dark cycle at a controlled room temperature (22 ± 0.5 °C) with unrestricted access to water and a standard diet (10% calories from fat) unless otherwise stated. All animal experiments were conducted according to the guidelines of the Institutional Animal Care and Use Committee of the Centre for Experimental Research in Clinical Medicine, Shengli Clinical Medical College of Fujian Medical University, Fuzhou, China (Permission Number: IACUC-FPH-PZ-20240624【0012】).

### Experimental design

**Fig. 1:**
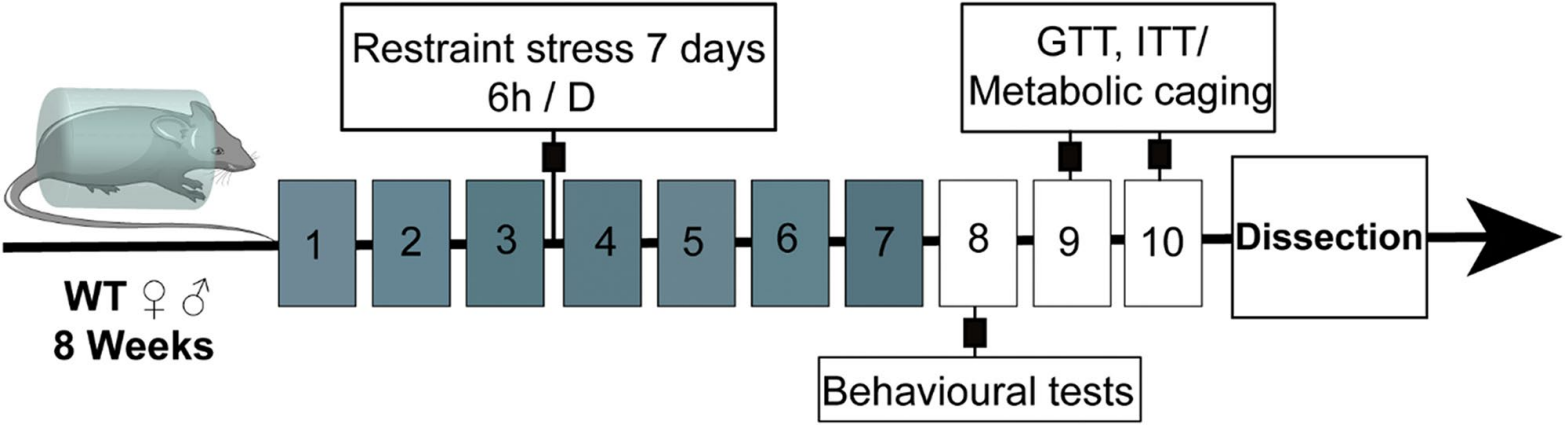
Experimental design. In this experiment, 8-week-old wild-type C57BL/6 mice were subjected to chronic restraint stress (CRS) for six hours per day for seven consecutive days. On the eighth day, behavioural tests were conducted. The mice were conducted in two batches, with one batch undergoing glucose tolerance tests (GTT) and insulin tolerance tests (ITT) on days nine and ten, while the other batch was subjected to metabolic cage analyses. At the end, mice were dissected for blood and adipose tissue samples

The CRS model was established by restraining mice in 50 mL plastic centrifuge tubes with ventilation holes for 6 h daily (8:00 a.m. to 2:00 p.m.) over seven consecutive days [29–31]. A fasting control group was included to account for the metabolic effects of food and water deprivation during the restraint period. Mice were randomly assigned to groups (15–18 mice per group, total *n* = 100). For the fasting and stress groups, mice were allowed ad libitum access to food except during the 6-hour restraint period. The study meticulously measured the daily food intake and body weight at 8:00 AM for seven consecutive days of restraint stress and calculated the total food consumption and body weight reduction. Following the restraint period, behavioural tests, glucose tolerance tests (GTT), insulin tolerance tests (ITT) and metabolic cage studies were performed in batches (Fig. 1). After the experiments ended, mice were deeply anaesthetised with pentobarbital (Sigma Aldrich, Missouri, USA). Blood samples were collected for plasma hormone analysis and adipose tissues were harvested. Tissues were flash-frozen in liquid nitrogen and stored at − 80 °C for subsequent analyses.

### Behavioural experiments

Stress-exposed mice underwent behavioural assessments, including the open field test (OFT), elevated plus maze (EPM) and tail suspension test (TST), to evaluate locomotor activity and depression-like behaviours. All tests were conducted in a dimly lit room, with mice acclimated overnight prior to testing. Experiments were performed between 8:00 a.m. and 6:00 p.m. to minimise circadian influences. After each test, the surfaces of the equipment were cleaned with 75% ethanol to eliminate odour trails.

#### OFT

Mice were individually placed at the centre of a square arena (50 × 50 × 50 cm) constructed from grey polyvinyl chloride and monitored for 5 min using an automated video tracking system. Locomotor activity was recorded, and the digitised movement data were analysed using the DigBehv animal behaviour analysis programme (Shanghai, China). The central area, defined as one-quarter of the arena’s total size, was used to measure depression-like behaviour by tracking the time spent and the number of entries into the central zone.

#### EPM

The EPM consisted of two open arms (10 × 30 cm) and two enclosed arms (10 × 30 × 20 cm), positioned opposite each other, with a central platform measuring 10 × 10 cm and elevated 40 cm above the ground. Mice were placed individually at the centre of the maze, facing an open arm, and their activity was recorded for 5 min [32]. The time spent in the open arms and the number of open arm entries were quantified using the Smart v3.0 system (Panlab, Barcelona, Spain) (Supplementary Fig. 1A–D).

#### TST

For the TST, each mouse’s tail was affixed to a hanging hook approximately 1 cm from the tip, suspending the mouse in an inverted position about 10 cm above the ground. Behaviour was recorded for 6 min, and the last 4 min of immobility time were analysed. The latency to the first instance of immobility was also calculated using the Smart v3.0 system (Panlab, Barcelona, Spain) to assess depression-like behaviour [33] (Supplementary Fig. 1E-H).

### Intraperitoneal glucose and insulin tolerance test

The GTT test was performed on mice of both sexes that were fasted for 12 h (7:00 p.m. to 7:00 a.m. the following day). Glucose at a dose of 2 g/kg of body weight was administered intraperitoneal injection. For ITT, mice were fasted for 6 h (8:00 a.m. to 2:00 p.m.) and received an intraperitoneal injection of insulin (Novo Nordisk, Copenhagen, Denmark) of 1U/kg of body weight. Blood glucose concentrations were determined at 15, 30, 60, 90, 120, and 180-minute time pointes after glucose or insulin injection using a glucometer from Roche (Basel, Switzerland) and calculated the area under the curve (AUC).

### Indirect calorimetry test

The metabolic parameters of mice were monitored using TSE PhenoMaster animal monitoring system (Hofheim, Germany) during the recovery phase following stress. Mice were acclimated for 24 h before measurements were taken. The oxygen consumption (VO2), exhaled carbon dioxide (VCO2), respiratory exchange ratio (RER) [34], energy expenditure (EE) [35] and food consumption, water intake of each mouse were determined for a 24-hour period. The RER and EE were calculated based on the VO2 and VCO2 data. Analysis of covariance (ANCOVA) was used to compare metabolic parameters of mice, in which body weight was used as covariate.

### Biochemical analyses of the plasma

The blood of mice in each group was collected from the medial canthus vein with stand for 4 h, then centrifuged at 3000 rpm for 15 min at 4 ℃, and taken out the supernatant. The serum level of corticosterone (CORT), luteinizing hormone (LH), follicle stimulating hormone (FSH) detected respectively by enzyme-linked immunosorbent assay (ELISA) kits from Meimian (Jiangsu, China) and testosterone (T), oestradiol (E2) detected by ELISA kit from Beyotime (Shanghai, China), listed in supplementary Table 1. The procedures were performed according to the manufacturer’s instructions.

### Hematoxylin and Eosin staining (H&E) of adipose tissues

Adipose tissues were fixed in 4% paraformaldehyde for 24 h, embedded in paraffin and sectioned at a thickness of 5 μm. Following deparaffinisation and rehydration, slides were stained with H&E and imaged using a Nikon Eclipse Ci-L microscope (Tokyo, Japan). Adipocyte counts and lipid droplet areas were quantified using AdipoCount software (Shanghai, China) in at least three fields per slide at 200× magnification [36].

### Quantitative real-time polymerase chain reaction (RT-PCR)

Samples were extracted using RNA isolater Total RNA Extraction Reagent (Vazyme, Jiangsu, China). Total RNA (1000 ng) was reverse transcribed to generate complementary DNA using PrimeScipt RT Reagent Kit with genomic DNA Eraser (Takara, Kusatsu, Japan). Quantitative RT-PCR was performed on CFX 96 Real-Time system (Bio-Rad, California, USA) using SYBR Premix Ex Taq II Kit (Takara, Kusatsu, Japan) with specific primers synthesized by Shangya (Fuzhou, China), listed in Supplementary Table 2. 𝛽 actin was used as an internal control for adipose tissues.

### Immunohistochemistry and Immunofluorescence

For immunohistochemistry, adipose tissues were fixed with 4% paraformaldehyde overnight, permeabilized with 0.2% Triton X-100 for 10 min, and blocked with 5% normal goat serum at room temperature for 60 min. After that, samples were incubated with anti-UCP1 (1:100, Cell Signaling Technology (CST), Massachusetts, USA) antibody at 4 °C overnight. The following day, images were collected following incubation with the secondary antibody and staining with diaminobenzidine (DAB) and haematoxylin (Supplementary Fig. 4G–H).

For immunofluorescence, paraffin embedded tissue slides were deparaffinised and subjected to antigen retrieval in citric acid buffer, blocked with 5% normal goat serum. Incubated with anti-GR (1:50, Santa Cruz, California, USA) or anti-UCP1 (1:100, CST, Massachusetts, USA) antibody at 4 °C overnight and then incubated with the corresponding secondary antibody at room temperature for 1 h, finally stained with diamidino-2-phenylindole (DAPI) 20 min. Images were captured using a Nikon Eclipse Ci-L microscope (Tokyo, Japan). The used primary antibodies were listed in Supplementary Table 3.

### Western blotting

Total protein was extracted and protein concentrations were measured using protein assay kits from Solarbio (Beijing, China). The following procedures were performed: total protein was separated and transferred to PVDF membranes (Millipore, Massachusetts, USA). After blocking the membrane with 5% bovine serum albumin (BSA, Beyotime, Shanghai, China), incubated the membrane with various primary antibodies (Supplementary Table 3) at 4 ℃ overnight and then incubated with secondary antibodies at room temperature for 30 min. Finally, protein detection was performed using a chemiluminescence instrument.

### Statistical analysis

Prior to analysis, data were subjected to normality tests. Differences between stressed mice and wild-type (WT) controls or fasting controls were evaluated using unpaired Student’s *t*-test for comparisons between two groups, ordinary one-way ANOVA with Bonferroni post hoc tests for comparisons among three or more groups, or two-way ANOVA with Bonferroni post hoc tests for interactions between factors, all performed in GraphPad Prism 9 (GraphPad Software, California, USA). Data were presented as mean ± standard error of the mean (SEM), and *P* < 0.05 was considered statistically significant.

## Results

### Mice subjected to CRS exhibit depression-like behavioural manifestations

**Fig. 2:**
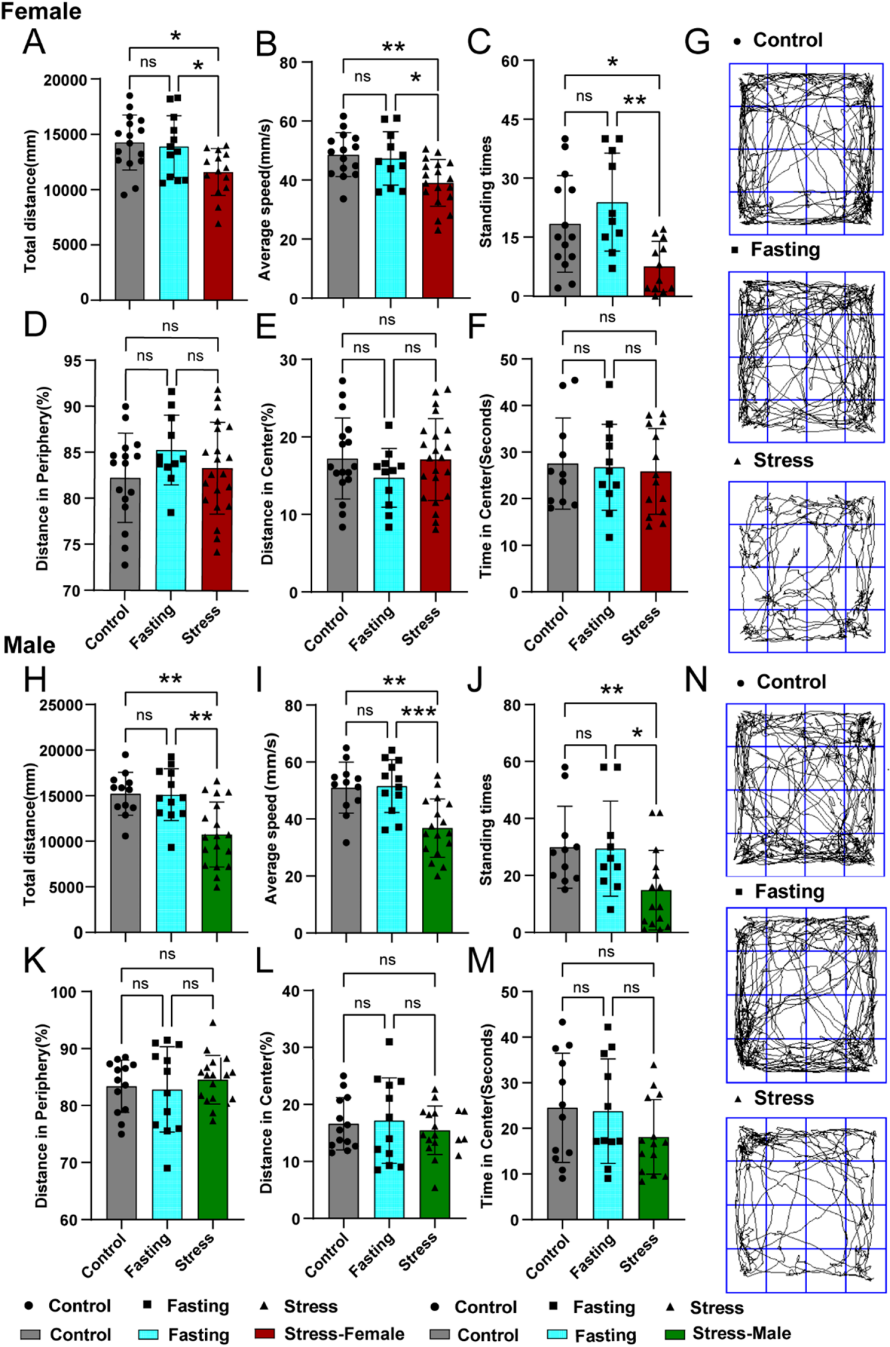
Behavioural experiments of mice under chronic restraint stress (CRS). Impact of chronic stress on behavioural changes in locomotor activity and depression-like behaviour for female mice in the open field area (**A**–**G**), and in male mice (**H**–**M**). For the open field test (OFT), total track distance for females (**A**) and in males (**H**), and average speed during the test for females (**B**) and in males (**I**), and the number of standing for females (**C**) and in males (**J**) were recorded. Percentage (%) of distance spent in the periphery zone for (**D**) females and (**K**) males, and percentage (%) of distance spent in the centre zone for (**E**) females and (**L**) males. Time spent in the centre zone (5 min total test time) for (**F**) females and (**M**) males, and track during the test for females (**G**) and in males (**N**). Data are expressed as the mean ± SEM (*n* = 15–23) as determined by one-way ANOVA with Bonferroni post hoc tests. *P < 0.05, **P < 0.01, and ***P < 0.001 for stress mice versus WT control or fasting control mice. Ns, means not significant. Error bars represent SEM

Behavioural and locomotor changes in CRS-exposed mice were assessed through the OFT (Fig. 2), EPM and TST (Supplementary Fig. 1). Stress-exposed mice demonstrated reduced track distance and average speed in the OFT (Fig. 2A–B, H–I), decreased exploratory behaviour (Fig. 2C, J). Even though there was no significant difference in the time spent in the central and peripheral areas (Fig. 2D–G, K–N). EPM testing revealed a reduction in time spent in the open arms, meanwhile TST showed prolonged immobility time in female mice (Supplementary Fig. 1A–H). Collectively, these results indicate depression-like behavioural phenotypes in CRS-exposed mice.

### CRS induced a lean phenotype and hormone disturbance in mice

**Fig. 3:**
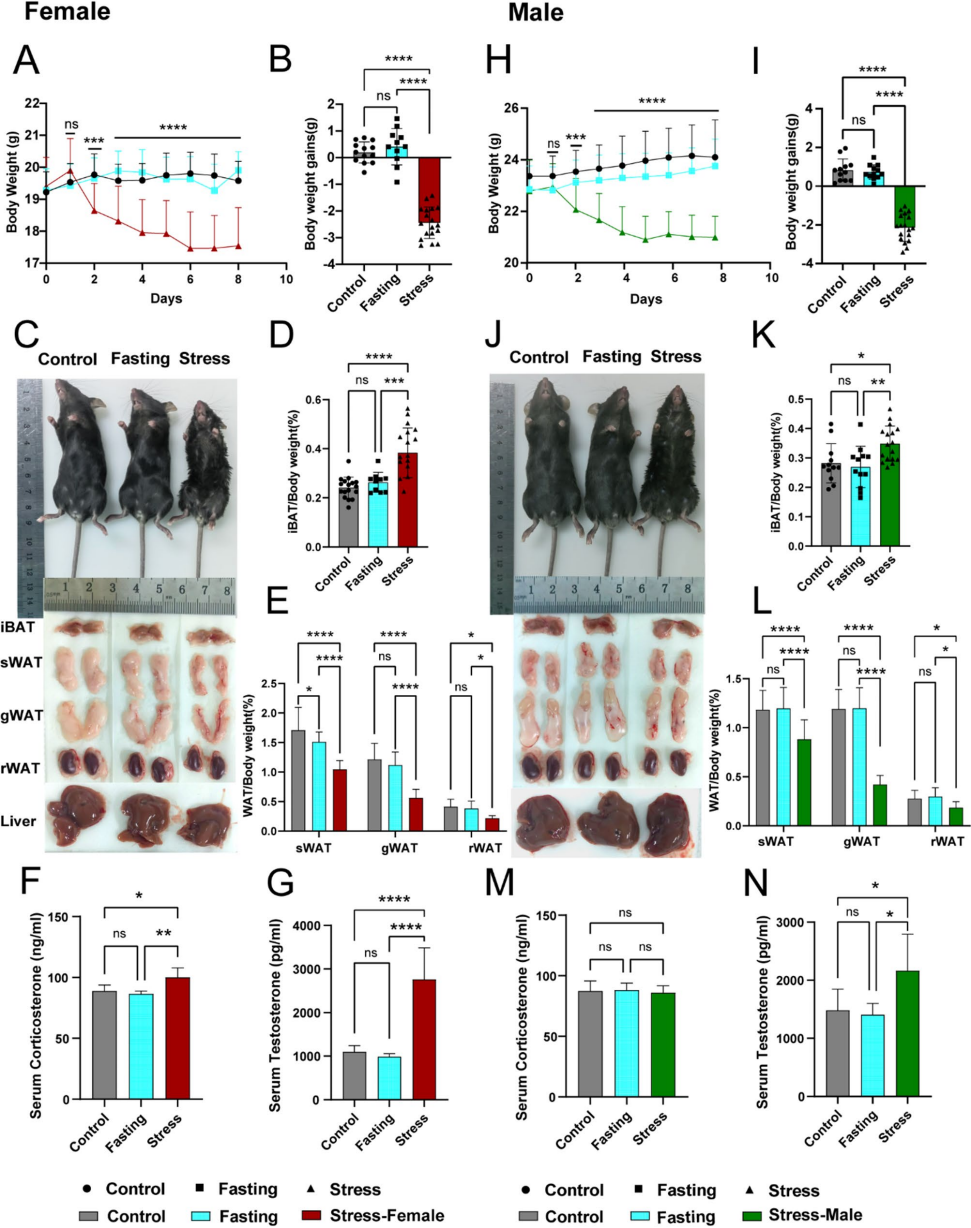
CRS induced a lean phenotype and hormone disturbance in mice. Time course of body weight in seven days’ restraint stress for females (**A**) and males (**H**), and final body weight gains (**B**,** I**). Appearance of representative mice after CRS versus normal diet and fasting stress, and their tissues for females (**C**) and males (**J**). Percentage (%) weights of dissected iBAT in females (**D**) and males (**K**), and sWAT, gWAT, rWAT weights in females (**E**) and males (**L**). All data are expressed as the mean ± SEM (*n* = 15–18). Serum corticosterone and testosterone levels in females (**F**,** G**) and males (**M**,** N**). Data are expressed as the mean ± SEM (*n* = 6–8). Data were determined by one-way ANOVA or two-way ANOVA with Bonferroni post hoc tests. *P < 0.05, **P < 0.01, ***P < 0.001 and ****P < 0.0001 for stress mice versus WT control or fasting control mice. Ns, means not significant. Error bars represent SEM

Mice exposed to CRS exhibited a negative energy balance. Over the 7-day restraint stress period, these mice consumed less food (Supplementary Fig. 2A–B) and experienced significant weight loss (Fig. 3A–B, H–I). Additionally, when restrained, the mice exhibited intense struggling and sweating due to the physical and psychological stress of confinement. These observations led to the conclusion that the mice were in a state of negative energy balance during the restraint stress period, with energy expenditure exceeding intake. Organ weights measured at the end of the experiment revealed contrasting phenotypes among adipose tissues: increased interscapular brown adipose tissue (iBAT) weight (Fig. 3D, K) and reduced weights of subcutaneous (sWAT), gonadal (gWAT) and retroperitoneal (rWAT) white adipose tissues (Fig. 3E, L). Moreover, serum corticosterone levels were elevated in female mice (Fig. 3F) but not in males (Fig. 3M). Testosterone levels significantly increased in both female and male mice (Fig. 3G, N). In male mice, LH and FSH levels decreased (Supplementary Fig. 2F–G), whereas no significant changes were observed in female mice (Supplementary Fig. 2C–E), possibly due to differences related to the oestrous cycle and sex-specific responses.

### CRS impairs insulin sensitivity and enhances RER, with carbon-based foods as the primary energy source

**Fig. 4:**
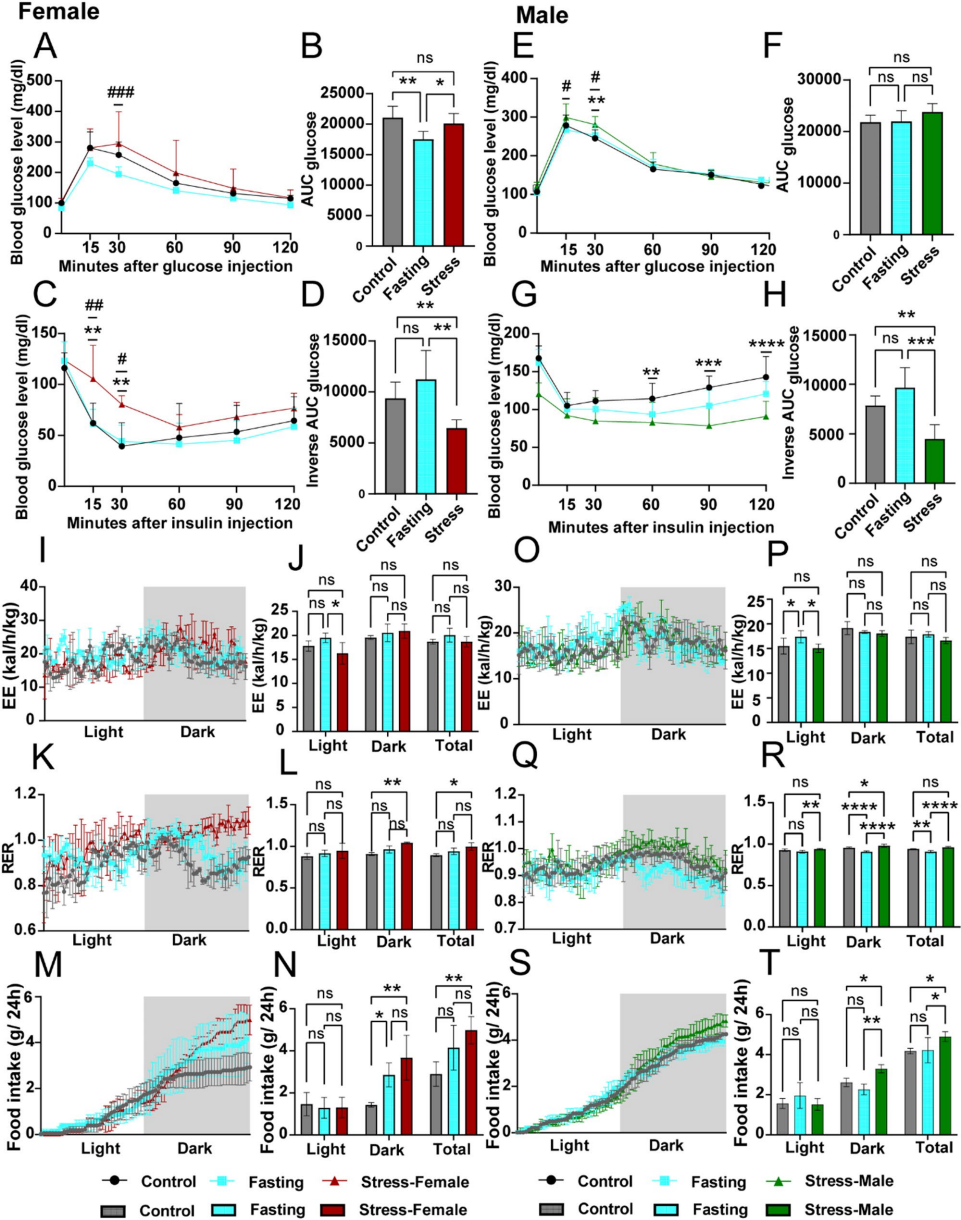
The insulin sensitivity of mice under CRS decreased, and the RER increased. The intraperitoneal glucose tolerance test (GTT) was performed on females (**A**) and males (**E**) (dose 2 g/kg), and the corresponding area under the curve (AUC) were calculated for females (**B**) and males (**F**). The intraperitoneal insulin tolerance test (ITT) was performed on females (**C**) and males (**G**) (dose 1U/kg). Area above the ITT curves for females (**D**) and males (**H**) (*n* = 5–8 mice per group). Energy expenditure (EE) and respiratory exchange ratio (RER) were determined by metabolic cages in females (**I**–**L**) and males (**O**–**R**). Cumulative food intake over 24 h also calculated for females (**M**–**N**) and males (**S**–**T**) (*n* = 3–6 mice per group). Data are expressed as the mean ± SEM and were determined by one-way ANOVA or two-way ANOVA with Bonferroni post hoc tests. In (A), (C), (E), (G), *#P* < 0.05, *##P* < 0.01, and *###P* < 0.001 for stress versus fasting mice; *P < 0.05, **P < 0.01, ***P < 0.001 and ****P < 0.0001 for stress mice versus control mice. Others, *P < 0.05, **P < 0.01, ***P < 0.001 and ****P < 0.0001 for stress mice versus WT control or fasting control mice. Ns, means not significant. Error bars represent SEM

In CRS-exposed mice, glucose tolerance remained largely unchanged, except for a higher glucose level at the 30-minute time point compared to the control group (Fig. 4A–B, E–F). Female fasting mice exhibited lower blood glucose levels (Fig. 4B). However, insulin sensitivity was markedly impaired in stress-exposed mice of both sexes, as evidenced by reduced glucose excursions (Fig. 4C, G) and a significantly lower inverse AUC compared to controls (Fig. 4D, H).

Metabolic cage analyses revealed that apart from the elevated oxygen consumption in the fasting group during the daytime (Supplementary Fig. 3A–B, G–H), no other significant differences were observed, and no significant changes in carbon dioxide production (Supplementary Fig. 3C-D, I–J) of stressed mice following seven days of CRS. Consequently, only the fasting group of mice exhibited an increase in total energy expenditure, while the restraint-stressed mice did not show any significant difference during the recovery phase following restraint stress (Fig. 4I–J, O–P). However, the RER significantly increased, particularly during the dark cycle (Fig. 4K–L, Q–R). There was also an increased food consumption (Fig. 4M–N, S–T) and water intake over 24 h (Supplementary Fig. 3E–F, K–L) of restraint-stressed mice during the recovery phase. These findings suggest that CRS-exposed mice predominantly relied on carbohydrates as their primary energy source to compensate for the reduction in WAT caused by excessive energy expenditure during the restraint stress period [35, 37]. The results indicate that, following adjustments in their energy-related behaviour and adaptive changes in fat metabolism, the mice achieved a state of adaptive energy balance. In contrast, male fasting mice exhibited lower RER values (Fig. 4Q–R) [38] and demonstrated slightly higher energy expenditure (Fig. 4O–P) [39, 40], which indicated the gender-specific differences in metabolic response.

### Mice exposed to CRS underwent significant adipose tissue remodelling as a metabolic stress adaptation

**Fig. 5:**
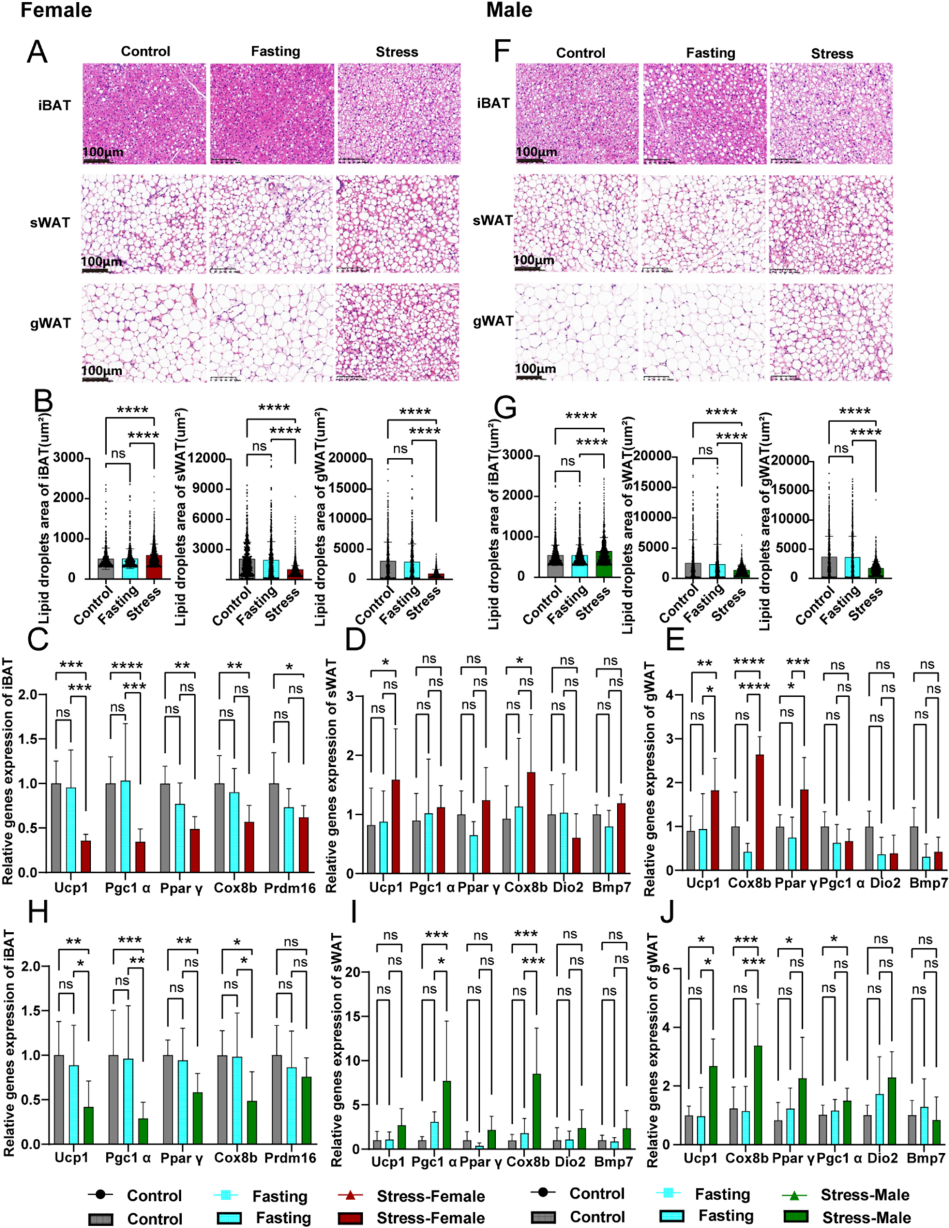
Mice exposed to CRS underwent significant adipose tissue remodelling. Representative H&E-staining images of iBAT, sWAT, and gWAT with 200× magnification from WT control, fasting and stress mice in females (**A**) and males (**F**). Distribution of lipid droplets area (µm²) in iBAT, sWAT, and gWAT in females (**B**) and males (**G**) (*n* = 4–6 mice per group). The mRNA expression levels of thermogenic genes were evaluated in iBAT, sWAT, and gWAT from females (**C**–**E**) and males (**H**–**J**) (*n* = 5–10 mice per group). Data are expressed as the mean ± SEM and were determined by one-way ANOVA or two-way ANOVA with Bonferroni post hoc tests. *P < 0.05, **P < 0.01, ***P < 0.001 and ****P < 0.0001 for stress mice versus WT control or fasting control mice. Ns, means not significant. Error bars represent SEM

Representative H&E staining of iBAT, sWAT and gWAT revealed significant changes in adipose tissue morphology in CRS-exposed mice (Fig. 5A, F). Adipose tissue mass is influenced by both the average size and the number of constituent adipocytes [41]. A notable browning effect was observed in WAT, characterised by a reduction in lipid droplet size (Fig. 5B, G) and an increase in the number of lipid droplets (Supplementary Fig. 4B–C, E–F). In contrast, BAT exhibited an accumulation of lipid droplets (Fig. 5B, G; Supplementary Fig. 4A, D), consistent with changes in tissue weight (Fig. 3C–E, J–L). Moreover, the expression of thermogenic genes, including *Ucp1*, *Peroxisome proliferator-activated receptor gamma coactivator 1-alpha* (*Pgc1α*), *Peroxisome proliferator-activated receptor gamma* (*Pparγ*) and *Cytochrome c oxidase subunit 8b* (*Cox8b*), was upregulated in WAT, particularly in gWAT (Fig. 5D–E, I–J). Conversely, these genes were significantly downregulated in BAT (Fig. 5C, H), indicating impaired thermogenesis.

### Western blotting analysis uncovers a correlation between GRs and thermogenic proteins, indicating their role in adipose tissue remodelling

**Fig. 6:**
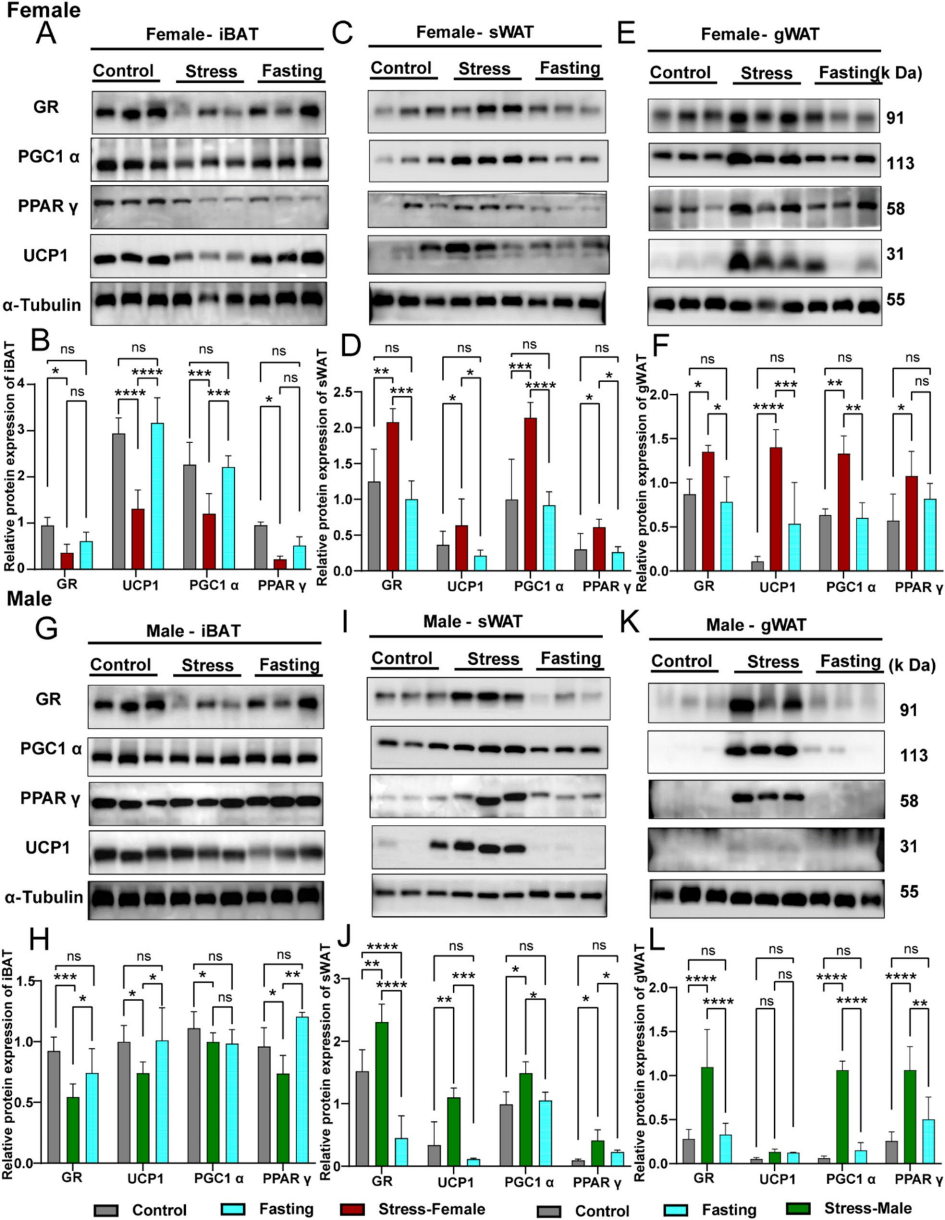
Western blotting analysis uncovers a correlation between GRs and thermogenic proteins. Western blotting analysis of the BAT from control, fasting and stress mice and densitometric quantification of the western blots normalized to α-Tubulin in females (**A**–**B**) and males (**G**–**H**). Western blotting analysis of the sWAT, gWAT and densitometric quantification of the western blots normalized to α-Tubulin in females (**C**–**F**) and males (**I**–**L**). Data are expressed as the mean ± SEM (*n* = 3) and were determined by two-way ANOVA with Bonferroni post hoc tests. *P < 0.05, **P < 0.01, ***P < 0.001 and ****P < 0.0001 for stress mice versus WT control or fasting control mice. Ns, means not significant. Error bars represent SEM

In chronic stress, the HPA axis is activated [42], and GRs play an important role in adipose tissue lipid metabolism [23, 43]. Therefore, this study examined GR protein expression in various adipose tissues and found it aligned with thermogenic protein expression in both BAT and WAT. Specifically, in the ‘whitening’ BAT of metabolically stressed mice, the expression of UCP1, PGC1α and PPARγ proteins was reduced, accompanied by a significant decrease in GR expression (Fig. 6A–B, G–H). Conversely, in the ‘browning’ sWAT and gWAT, UCP1, PGC1α and PPARγ protein levels were elevated correlating with increased GR expression (Fig. 6C–E, I–L). Immunohistology analysis of UCP1 protein in BAT and gWAT tissues mirrored the trends observed in mRNA expression (Supplementary Fig. 4G–H). GR expression levels were inversely correlated in BAT and WAT, but aligned with the expression patterns of thermogenic proteins across adipose tissues. These findings suggest that under chronic metabolic stress, GRs mediate tissue-specific responses in adipose tissues, driving functional and phenotypic transitions in BAT and WAT to maintain energy homeostasis.

### Downregulation of UCP1 and GR in ‘whitening’ BAT accompanied by organelle dysfunction

**Fig. 7:**
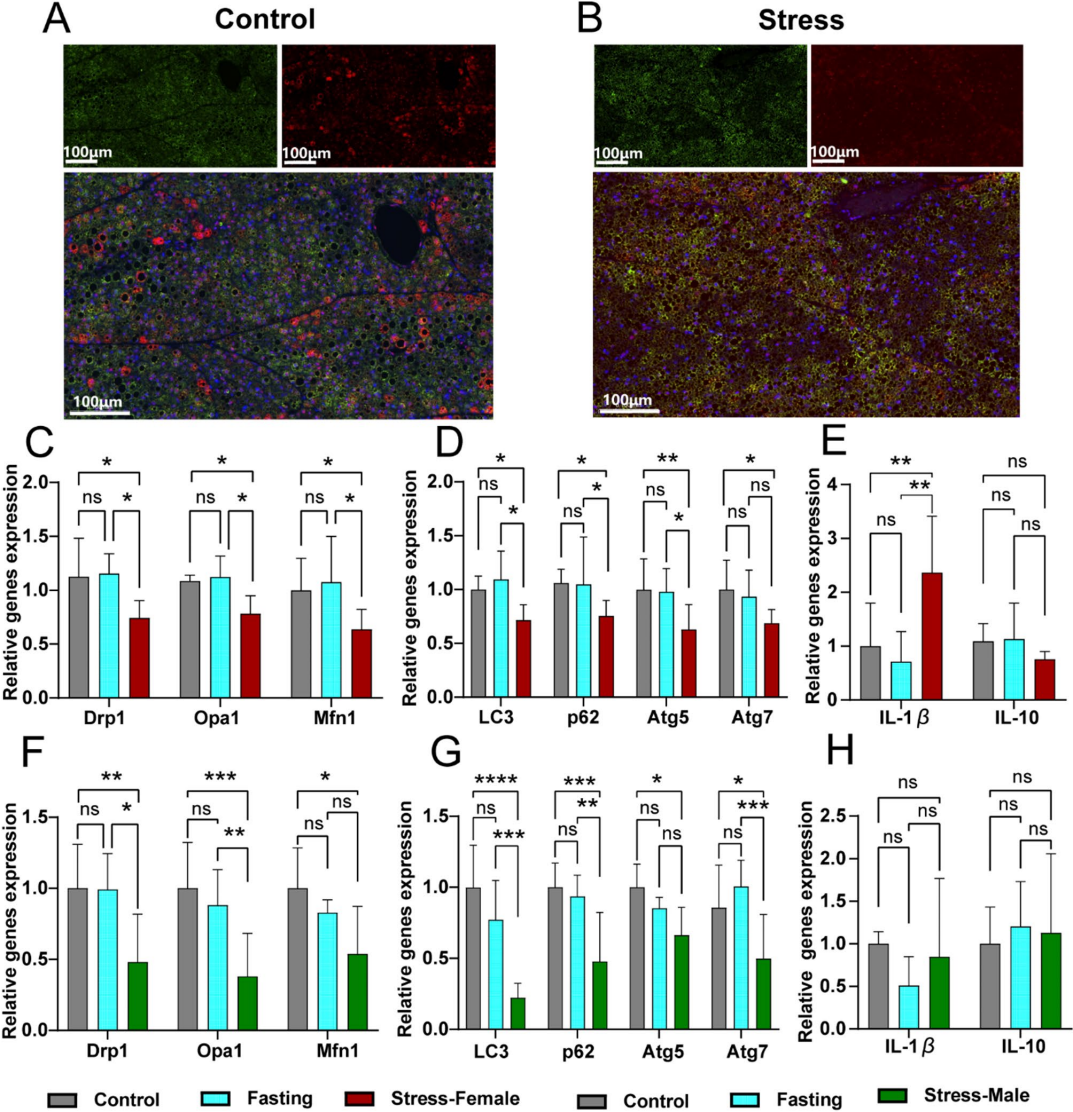
Brown adipose underwent ‘whitening’, which was accompanied by organelle dysfunction. Representative images of immunofluorescence for UCP1 (green), GR (red), and nuclei (blue) in brown adipocytes with 200× magnification from control mice (**A**) and stress mice (**B**). The mRNA expression levels of mitochondrial function-related genes, autophagy-related genes and inflammatory-related genes were evaluated in females (**C**–**E**) and males (**F**–**H**). Data are expressed as the mean ± SEM (*n* = 4–9) and were determined by two-way ANOVA with Bonferroni post hoc tests. *P < 0.05, **P < 0.01, ***P < 0.001 and ****P < 0.0001 for stress mice versus WT control or fasting control mice. Ns, means not significant. Error bars represent SEM

Furthermore, immunofluorescence colocalization assays further demonstrated that depolarised BAT in stressed mice exhibited a significant reduction in cytoplasmic GRs (Fig. 7A–B). As reported, the process of BAT ‘whitening’ has been associated with both autophagy [44] and mitochondrial dysfunction [45]. To explore the mechanisms underlying the ‘whitening’ phenotype of BAT in stressed mice, the study identified mitochondrial dysfunction, evidenced by reduced mRNA expression of key mitochondrial dynamics regulators, including *Dynamin-related protein 1* (*Drp1*), *Optic atrophy 1* (*Opa1*) and *Mitofusin 1* (*Mfn1*) (Fig. 7C, F). Autophagy was also impaired, as indicated by decreased mRNA expression of autophagy-related genes, such as *Microtubule-associated protein light chain 3* (*LC3*), *Sequestosome 1* (*p62*), *Autophagy-related protein 5* (*Atg5*) and *Autophagy-related protein 7* (*Atg7*) (Fig. 7D, G). Additionally, inflammation was exacerbated, with increased levels of *Interleukin-1β* (*IL-1β*) observed in female mice (Fig. 7E) but in male mice (Fig. 7H). These alterations may be linked to the reduced expression of GR, which could further aggravate the dysfunctional phenotype of BAT under chronic stress conditions.

### Fasting exerts a subtle influence on metabolic shifts in mice under CRS

CRS significantly affected mice, leading to alterations in emotional behaviour, body weight and adipose tissue remodelling. Both female and male mice exhibited consistent outcomes, although some results showed gender-specific responses. Notably, a comprehensive analysis of the majority data indicated that the phenotypes of the fasting control group were largely aligned with those of the blank control group, while both groups displayed pronounced metabolic differences compared to the restraint stress group. It should be noted that the restraint stress model inherently includes fasting as one of the stressors, and the certain impact of fasting on the metabolic changes caused by restraint stress cannot be denied. Overall, the phenotypic changes observed during the restraint stress period were not primarily attributable to fasting or water deprivation.

## Discussion

Adipose tissue remodelling is influenced by various exogenous and endogenous factors, including energy status fluctuations and hormonal changes [22] and is closely associated with the pathophysiology of metabolic disorders. Once regarded merely as a passive lipid reservoir, adipose tissue is now recognised as a metabolically active organ playing a central role in whole-body energy homeostasis. It contributes to critical processes such as immune responses, glucose metabolism, insulin sensitivity and thermogenesis [19]. While lipolysis is known to fulfil energy demands during negative energy balance [7], the mechanisms underlying adipose tissue remodelling in states of emaciation remain poorly understood.

Auger C et al. highlight the heterogeneity of adipose tissue, emphasising its regional variations in metabolic processes and hormonal responses [22]. This emerging perspective has drawn significant attention. The present study primarily investigated the pathophysiological implications of adipose tissue metabolism and associated mechanisms under CRS.

BAT, specialised for energy expenditure, is pivotal in maintaining energy homeostasis through adaptive thermogenesis [18]. This study demonstrated that mice subjected to CRS exhibited a pronounced ‘whitening’ phenotype in BAT, coupled with impaired thermogenic function. This may be attributed to the intense physical struggle during restraint, which likely triggered a substantial increase in skeletal muscle shivering thermogenesis. Consequently, non-shivering thermogenesis in BAT was reduced, thereby decreasing BAT’s energy consumption for thermogenesis. These findings align with prior research, suggesting that individuals with anorexia nervosa experience a compensatory reduction in basal metabolic rate to mitigate chronic energy deficits caused by restrictive eating behaviours [46, 47]. Furthermore, the study revealed that chronic and excessive GC exposure contributes to lipid accumulation in specific body regions, particularly in BAT. This GC-induced lipid deposition promotes a ‘whitening’ phenotype in BAT, impairing its thermogenic capability [48, 49].

WAT plays a crucial role in regulating various physiological processes that impact energy balance. It is also a significant target for other organs, contributing to energy homeostasis by managing the storage and utilisation of fatty acids [17]. This study demonstrated that under conditions of chronic stress and substantial energy expenditure, the mobilisation of glucocorticoid hormones for adipose remodelling in mice serves a protective role against metabolic stress. Both sWAT and gWAT participated in the browning process, compensating for BAT dysfunction to maintain energy homeostasis. These findings align with the research of Schulz et al. [50] and Wu et al. [51]. Furthermore, a notable reduction in fat mass among stressed mice led to an increase in the RER, with carbohydrates becoming the primary energy substrate. Conversely, fasting male mice exhibited a decrease in RER due to restricted dietary intake. As a pivotal endocrine tissue, WAT secretes various hormones and inflammatory factors and expresses multiple receptors for insulin, leptin, steroid hormones (e.g., glucocorticoids, androgens, oestrogens) and catecholamines [52], enabling homeostatic regulation.

Chronic stress is widely recognised to activate the HPA axis [42], leading to GCs release, which reallocates energy reserves to meet immediate or anticipated demands. This study highlights the interaction between GRs and adipose remodelling, suggesting that GRs specific-changes related to inducing insulin resistance, regulating lipids metabolism, and exacerbating inflammation. In general, chronic stress results in heightened HPA axis activity and diminished hypothalamic-pituitary-gonadal (HPG) axis activity. The elevated serum GC levels were observed in female mice, whereas no significant differences were detected in males. This discrepancy may be attributed to circadian fluctuations of the HPA axis and inconsistent blood collection times. Regarding reproductive hormones, both sexes exhibited significantly increased testosterone levels. However, other reproductive hormones were reduced in males but not in females, potentially due to the hormonal fluctuation characteristic of the female oestrous cycle. In this stress model, the gender-specific differences in metabolic response to chronic stress were also demonstrated in several indices. Hormones significantly influence body fat [53]. This study revealed increased levels of glucocorticoids and testosterone, both of which are known lipolytic hormones [54, 55]. Although GRs are nearly ubiquitous throughout the body, GCs exert cell- and tissue-specific effects [56]. In this study, the observed differential activity of the GRs across various adipose tissues has led to distinct adipose tissue-specific changes, resulting in phenotypic and functional transformations. These findings not only underscore the tissue-specific expression characteristics of GR but also reflects the dual role of the GC-GR axis in lipolysis and lipid accumulation. Moreover, stress models can result in distinct adipose phenotypes. For instance, Rebuffé-Scrive et al. observed an increase in mesenteric fat pad mass without changes in the epididymal, retroperitoneal, or inguinal regions in their stress model [57], contrasting with the findings in the current model. Meanwhile, testosterone, a fat-reducing hormone, appears to have a more pronounced effect on visceral adipose tissue [55], which also showed significant increases in this study.

The classic and well-established mechanism for enhancing thermogenesis in organisms involves the activation of β-adrenergic receptors (AR) by sympathetic nerve signals. This activation leads to an upregulation of PGC1α-driven mitochondrial biogenesis, while UCP1 facilitates the decoupling of the electron transport chain. This process generates heat and increases energy expenditure, further amplified by proton conductance using long-chain fatty acids derived from lipolysis [19]. Individuals exposed to chronic stress often experience dysregulation of the HPA axis, accompanied by heightened secretion of glucocorticoids and catecholamines. GCs enhance the lipolytic response in WAT to various hormones. Previous research has demonstrated that GRs are essential for signal transduction from β-AR to adenylate cyclase, leading to the activation of lipolysis [58]. These findings align with the diminished ability of GR-lacking adipocytes to undergo lipolysis under postprandial and fasting states, as a consequence of disrupted signalling from β-AR to adenylate cyclase, as elucidated by Mueller et al. [42]. In this study, the alterations in GR expression within adipose tissue were consistent with the expression of thermogenic proteins, suggesting that GRs play a key role in mediating fat remodelling in functional adipose tissue.

The process of BAT ‘whitening’ has been associated with both autophagy [44] and mitochondrial dysfunction [45]. Given the substantial body of research on the mechanisms underlying the whitening of BAT, this study conducted preliminary explorations. To further elucidate the potential mechanisms underlying the dysfunction of ‘whitening’ BAT in this stress model, the mRNA expression of genes related to mitochondria and autophagy was analysed. The results revealed mitochondrial dysfunction in ‘whitening’ BAT, accompanied by impaired autophagy. This impairment may contribute to excessive lipid accumulation in BAT [59], ultimately leading to impaired thermogenesis. Additionally, the reduction in GR expression may trigger an increase in inflammatory factor expression, potentially exacerbating this dysfunction. However, these findings require further in-depth investigation to be confirmed.

In addition to severe mental disorders associated with chronic stress, such as major depressive disorder (MDD) and post-traumatic stress disorder (PTSD), individuals subjected to extreme metabolic challenges under chronic stress, including earthquake survivors trapped in rubble, patients suffering from severe infections or undergoing major surgery in intensive care units, all experience psychological and physiological stressors that are comparable to those encountered in animal model. These insights have significant implications for understanding the metabolic consequences of chronic stress in humans and could inform the development of novel therapeutic approaches for metabolic disorders. While the CRS model provides valuable insights into the effects of chronic stress, it is important to note that it is an animal model and may not fully replicate the complexity of human stress responses.

### Study strengths and limitations

**Fig. 8:**
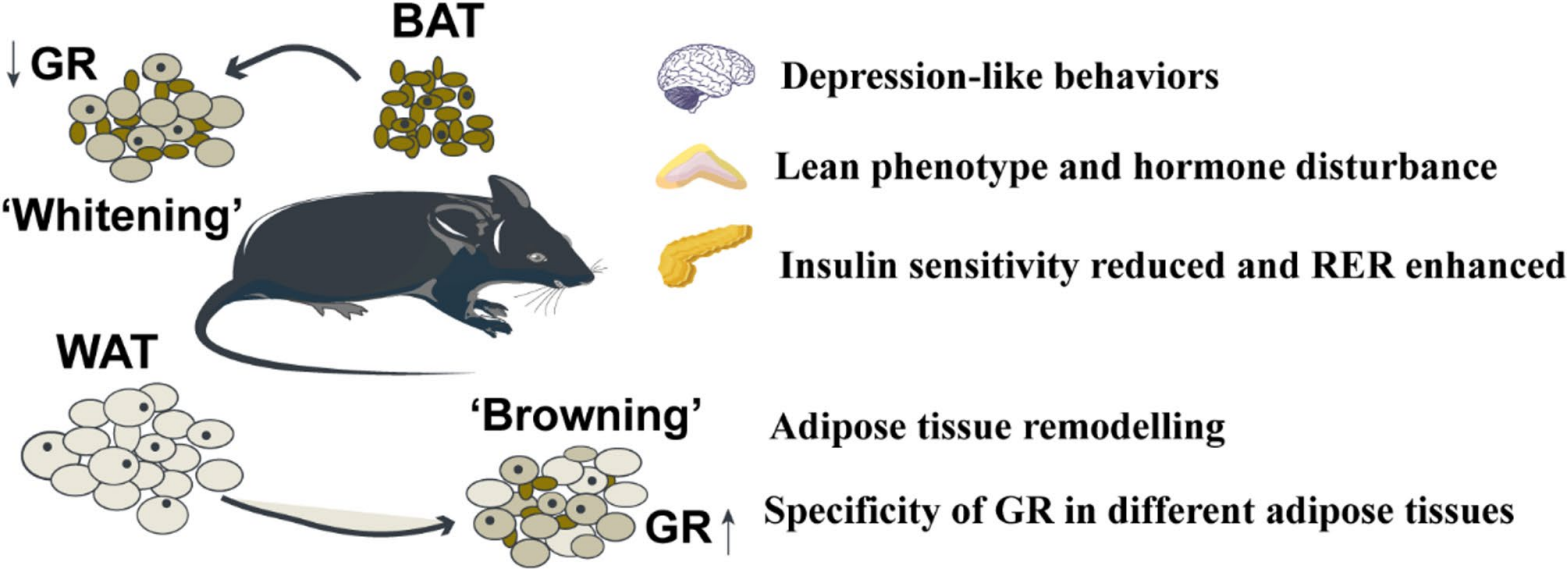
Overview of prominent effects as consequence of CRS on mice

This study provides a comprehensive evaluation of the physiological and metabolic alterations induced by CRS in mice. These include changes in emotional behaviour, body composition, glucose metabolism, metabolic state and, most notably, the functional remodelling of different adipose tissues along with their underlying mechanisms. The findings underscore the critical yet distinct roles of GR in adipocytes in maintaining lipid metabolic homeostasis, depending on the specific type of adipose tissue and its energetic state under CRS. The most prominent effects are summarised in Fig. 8.

Despite its strengths, this study has several limitations. While it provides valuable preliminary insights into the mechanisms driving BAT dysfunction, it does not delve deeply. Further research is needed to focus on identifying the specific intermediaries or signalling pathways involved in modulating GR activity in different adipose tissues to accomplish the ‘whitening’ and ‘browning’ processes. Additionally, the findings obtained from the mouse model are challenging to fully reflect the metabolic alterations and fat remodelling observed in patients with cachexia resulting from chronic stress.

## Conclusions

This study highlights the significant impact of CRS on adipose tissue remodelling in mice, characterised by a pronounced ‘whitening’ phenotype in BAT, with impaired thermogenic function, and a significant ‘browning’ response in various WAT depots. These findings suggest that the ‘browning’ response in WAT may serve as a protective adaptation to metabolic stress. The GRs within adipocytes play a pivotal role in this process by regulating systemic fuel partitioning and energy metabolism. The insights gained from this research deepen our understanding of the metabolic disruptions caused by chronic psychological and physiological stress and identify potential regulatory role of GRs in the thermogenic function of adipose tissue. These insights have significant implications for understanding the metabolic consequences of chronic stress in humans and could inform the development of novel therapeutic approaches for metabolic disorders. Further research is needed to unravel the complex mechanisms involved and to translate these findings into clinical practice.

## Electronic supplementary material

Below is the link to the electronic supplementary material.

Supplementary Material 1: Supplementary material 1: Table S1: List of used ELISA kits. Table S2: Specific primers of genes. Table S3: List of used primary anti-bodies for Western blotting, immunofluorescence and immunohistochemistry. Figure S1: EPM and TST tests of female mice also showed depression-like behaviours. Figure S2: During restraint stress, total food intake decreased, and reproductive hormones did not change significantly of females. Figure S3: During the recovery period after chronic restraint stress, there is no significant energy expenditure. Figure S4: The number of lipid droplets changed significantly, and the immunohistochemistry of protein expression of UCP1 in BAT and WAT. Figure S5. A certificate of language editing; Figure S6. All the raw data for uncorrupted western blotting.

Supplementary Material 2: The proof report of the overall similarity index of the MS on iThenticate.

## Data Availability

No datasets were generated or analysed during the current study.

## Abbreviations

CRS: Chronic Restraint Stress
BAT: Brown Adipose Tissue
WAT: White Adipose Tissue
UCP1: Uncoupling Protein-1
GC: Glucocorticoid
GR: Glucocorticoid Receptor
HPA: Hypothalamic–Pituitary–Adrenal
GTT: Glucose Tolerance Test
ITT: Insulin Tolerance Test
iBAT: Interscapular Brown Adipose Tissue
sWAT: Subcutaneous White Adipose Tissue
gWAT: Gonadal White Adipose Tissue
rWAT: Retroperitoneal White Adipose Tissue
OFT: Open Field Testl
EPM: Elevated Plus Maze
TST: Tail suspension Test
AUC: Area Under the Curve
VO2: Oxygen Consumption
VCO2: Exhaled Carbon Dioxide
RER: Respiratory Exchange Ratio
EE: Energy Expenditure
ANCOVA: Analysis of Covariance
CORT: Corticosterone
T: Testosterone
LH: Luteinizing Hormone
FSH: Follicle Stimulating Hormone
ELISA: Enzyme-linked Immunosorbent Assay
E2: Estradiol
H&E: Hematoxylin and Eosin
RT-PCR: Real-time Polymerase Chain Reaction
GAPDH: Glyceraldehyde 3-Phosphate Dehydrogenase
DAB: Diaminobenzidine
DAPI: Diamidino-2-Phenylindole
BAS: Bovine Serum Albumin
WT: Wild-Type
SEM: Standard Error of the Mean
Pgc1α: Peroxisome proliferator-activated receptor gamma coactivator 1-alpha
Pparγ: Peroxisome proliferator-activated receptor gamma
Cox8b: Cytochrome c oxidase subunit 8b
Drp1: Dynamin-related protein 1
Mfn1: Mitofusin1
Opa1: Optic atrophy 1
LC3: Microtubule-associated protein Light Chain 3
P62: Sequestosome 1
Atg5: Autophagy-related protein 5
Atg7: Autophagy-related protein 7
IL-1β: Interleukin-1β
HPG: Hypothalamic-Pituitary-Gonadal
AR: Adrenergic Receptor
