# Supplementary material for: Different action of glucocorticoid receptor in adipose tissue remodelling to modulate energy homeostasis by chronic restraint stress
Source: Lipids Health Dis. 2025 Mar 27;24:121. doi: 10.1186/s12944-025-02539-0 (PMC11948944; doi:10.1186/s12944-025-02539-0)
Supplement: Supplementary file 1 — Supplementary Material 1: Supplementary material 1: Table S1: List of used ELISA kits. Table S2: Specific primers of genes. Table S3: List of used primary anti-bodies for Western blotting, immunofluorescence and immunohistochemistry. Figure S1: EPM and TST tests of female mice also showed depression-like behaviours. Figure S2: During restraint stress, total food intake decreased, and reproductive hormones did not change significantly of females. Figure S3: During the recovery period after chronic restraint stress, there is no significant energy expenditure. Figure S4: The number of lipid droplets changed significantly, and the immunohistochemistry of protein expression of UCP1 in BAT and WAT. Figure S5. A certificate of language editing; Figure S6. All the raw data for uncorrupted western blotting. [file 12944_2025_2539_MOESM1_ESM.docx]

| **Genes** | **Primers Forward (5’ to 3’)** | | **Reverse (5’ to 3’)** | |
| --- | --- | --- | --- | --- |
| *Ucp1* | | GAGGTGTGGCAGTGTTCATT | | CTGTGGTGGCTATAACTCTGTAAG |
| *Pgc1α* | | CGCTGCTCTTGAGAATGGATAT | | GTCATACTTGCTCTTGGTGGAA |
| *Pparγ* | | ATGTCTCACAATGCCATCAGGT | | TTCCTGTCAAGATCGCCCTC |
| *Cox8b* | | TTCCCAAAGCCCATGTCTCT | | CTGGAACCATGAAGCCAACG |
| *Prdm16* | | ACCTGAAGAAGCACGAACAC | | TCGGATCTCGGAGAAGTAAGAAT |
| *Dio2* | | AATTATGCCTCGGAGAAGACCG | | GGCAGTTGCCTAGTGAAAGGT |
| *BMP7* | | TCGATACCACCATCGGGAGT | | GTCCAGCAAGAAGAGGTCCG |
| *Drp1* | | CAGCGAGATTGTGAGGTTATTG | | CGGATTCAGTCAGAAGGTCATC |
| *Opa1* | | GCTGGAATGCAATGACGTGG | | GAACTCGTTTGCCAGTGAGC |
| *Mfn1* | | AAGAGGACATTGTGTTTCGGTTT | | GGTTAGAAGGAGCAGTAGGAGTT |
| *LC3* | | TTCTTCCTCCTGGTGAATGG | | ATTGCTGTCCCGAATGTCTC |
| *p62* | | CTGTGGTGGGAACTCGCTAT | | AAGGGGTTGGGAAAGATGAG |
| *Atg5* | | GGAGAGAAGAGGAGCCAGGT | | GCTGGGGGACAATGCTAAT |
| *Atg7* | | TTTCCAGTCCGTTGAAGTCC | | GGACAATCTGGGCTAAATGC |
| *IL-1β* | | GAAATGCCACCTTTTGACAGTG | | TGGATGCTCTCATCAGGACAG |
| *IL-10* | | CCTGGGTGAGAAGCTGAAGAC | | CTTGTAGACACCTTGGTCTTGG |
| *β-actin* | | GCACCACACCTTCTACAATGA | | GCACCTACTTAATACACACTCCAA |
| *GAPDH* | | TGGAAAGCTGTGGCGTGATG | | TACTTGGCAGGTTTCTCCAGG |

**Supplementary Materials for**

Different action of glucocorticoid receptor in adipose tissue remodeling to modulate energy homeostasis by chronic restraint stress

**Supplementary Table 1. List of used** **ELISA kits.**

| **Product name** | **Information** | **Provider** |
| --- | --- | --- |
| Corticosterone ELISA Kit | Cat #MM-0061M1 | Meimian (Jiangsu, China) |
| Luteinizing hormone ELISA Kit | Cat #MM-44039M1 | Meimian (Jiangsu, China) |
| Follicle stimulating hormone ELISA Kit | Cat #MM-45654M1 | Meimian (Jiangsu, China) |
| Testosterone ELISA Kit | Cat #PT872; | Beyotime (Shanghai, China) |
| Estradiol ELISA | Cat #PE223 | Beyotime (Shanghai, China) |

**Supplementary Table 2. Specific primers of genes.**

**Supplementary Table 3. List of used primary antibodies for Western blotting, immunofluorescence and immunohistochemistry**

| **Antibodies** | **MW**  **kDa** | **Dilution** | **Provider** |
| --- | --- | --- | --- |
| Rabbit polyclonal anti-UCP1 (WB); Cat#23673-1-AP; RRID: AB_2828003 | 30 | 1:1000 | Proteintech (Wuhan, China) |
| UCP1 (E9Z2V) XP® Rabbit mAb Cat#72298T; RRID: AB_2862726 (IHC, IF); | 30 | 1:100 | Cell Signaling Technology (Massachusetts, USA) |
| PGC1α/β Rabbit mAb (WB); Cat#A19674; RRID: AB_2768318 | 113 | 1:1000 | ABclonal (Wuhan, China) |
| Rabbit polyclonal anti-PPARγ (WB); Cat#A11183; RRID: AB_2758449 | 53 | 1:1000 | ABclonal (Wuhan, China) |
| Glucocorticoid receptor polyclonal antibody (WB); Rabbit; Cat#24050-1-AP; RRID: AB_2813890 | 95 | 1:1000 | Proteintech (Wuhan, China) |
| Glucocorticoid Receptor antibody (G-5): sc-393232 (IF); RRID: AB_2687823 | 95 | 1:50 | Santa Cruz Biotechnology (California, USA) |
| α-Tubulin Mouse mAb (WB); Cat#AC012; RRID: AB_2768341 | 50 | 1:5000 | ABclonal (Wuhan, China) |


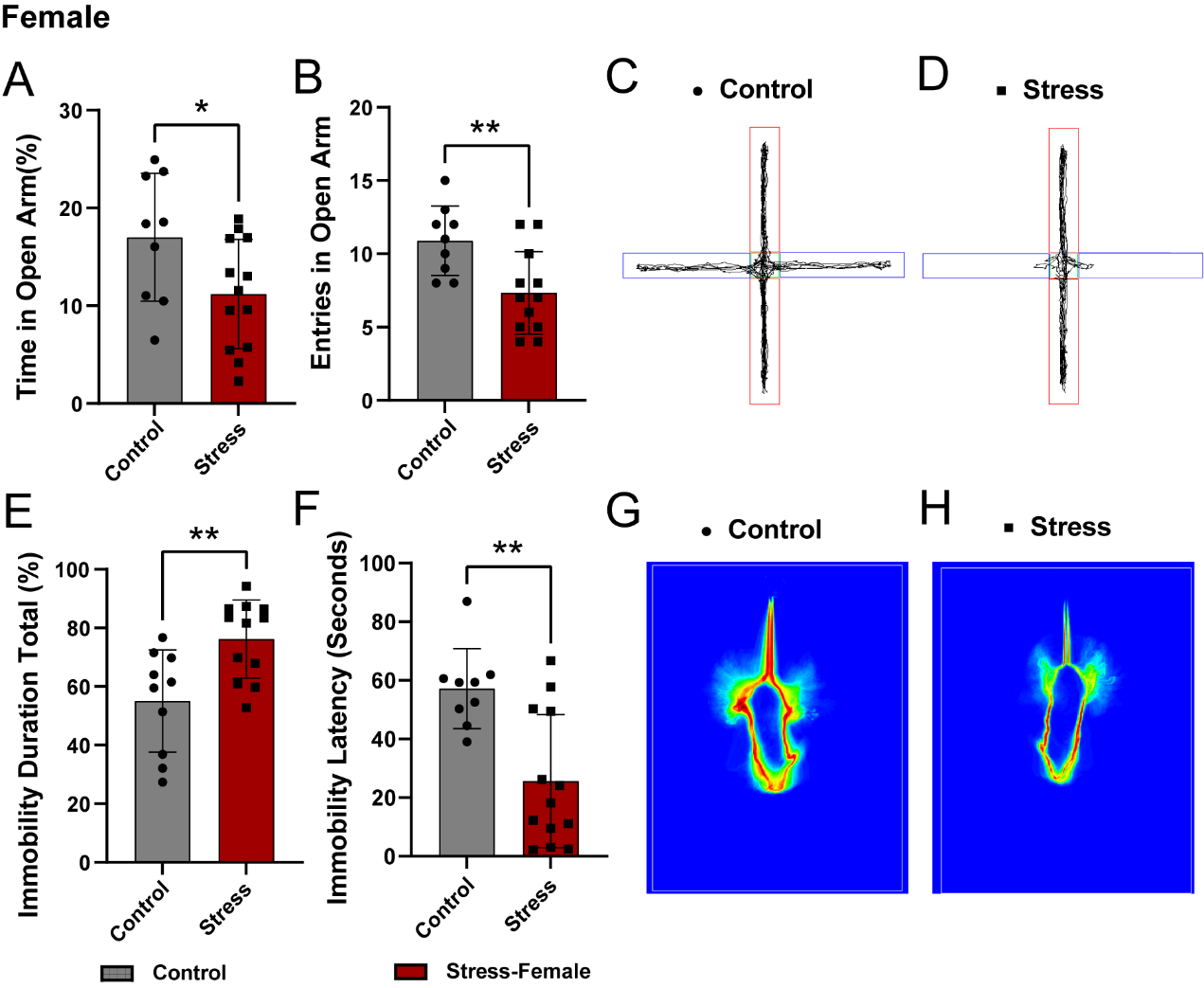


**Supplementary Figure 1. EPM and TST tests of female mice also showed depression-like behaviors.**

For the elevated plus maze (EPM) tests, percentage (%) of time spent in open arms (5 min total test time) for female mice (**A**) and frequency of open arms visits (**B**), and the track during the test for control mice (**C**) and stress mice (**D**). For the tail suspension tests, percentage (%) of last four minutes immobility time (6 min total test time) for female mice (**E**) and latency to first immobility (**F**), and the heat map of control mice (**G**) and stress mice (**H**). Data are expressed as the mean ± SEM (*n*=9-13). Statistical analysis was performed using Student’s t test. **P*<0.05, ***P*<0.01 for stress mice versus WT control mice. Error bars represent SEM.


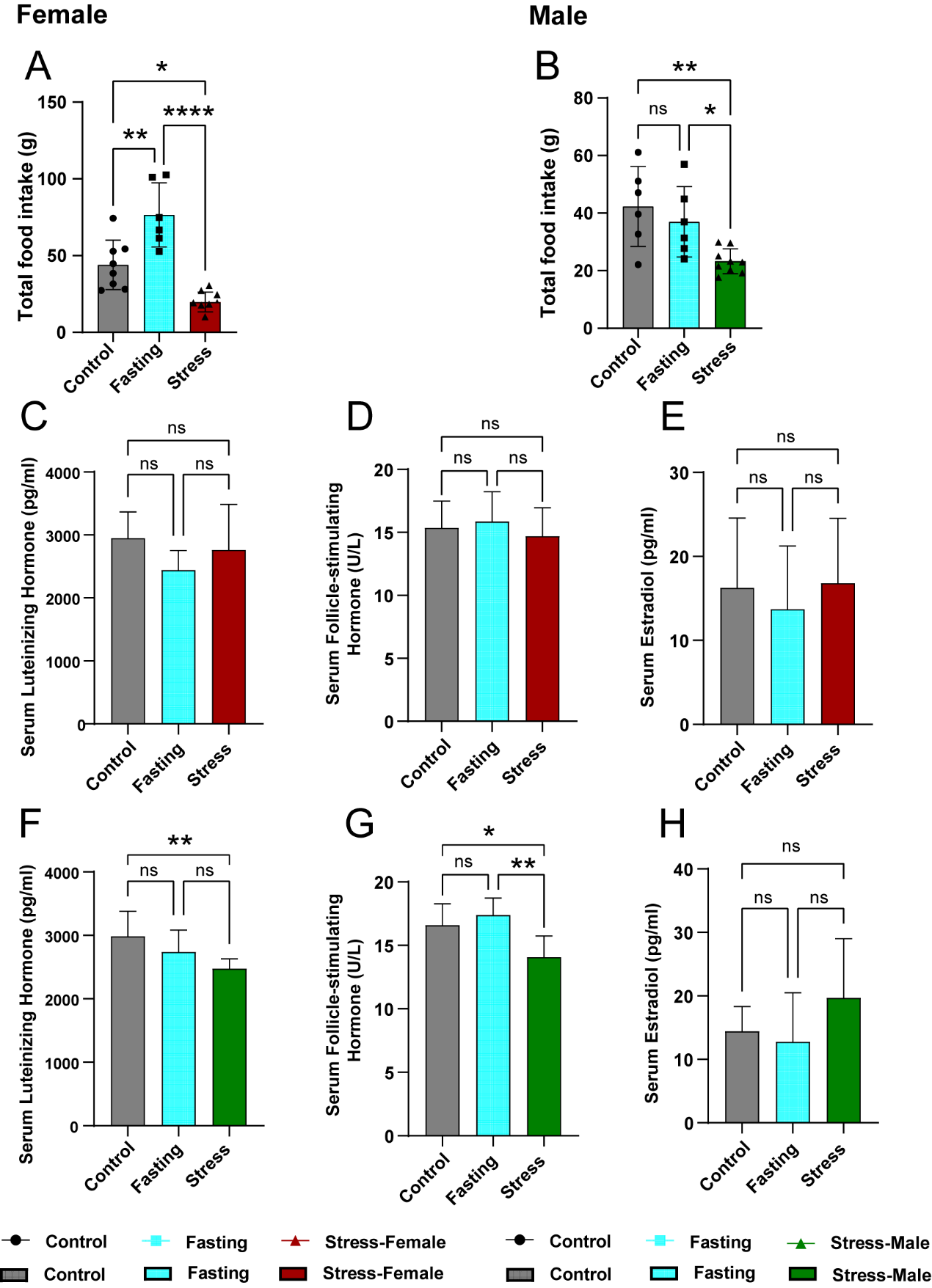


**Supplementary Figure 2. During restraint stress, total food intake decreased, reproductive hormones did not change significantly of females.**

Total food intake during the 7 days of restraint stress for females (**A**) and males (**B**). Serum luteinizing hormone, follicle stimulating hormone and estradiol levels in females (**C-E**) and males (**F-H)**. Data are expressed as the mean ± SEM (*n*=6-8). Data were determined by one-way ANOVA with Bonferroni post hoc tests. **P*<0.05, ***P*<0.01, ****P*<0.001 and *****P*<0.0001 for stress mice versus WT control or fasting control mice. Ns, means not signiﬁcant. Error bars represent SEM.


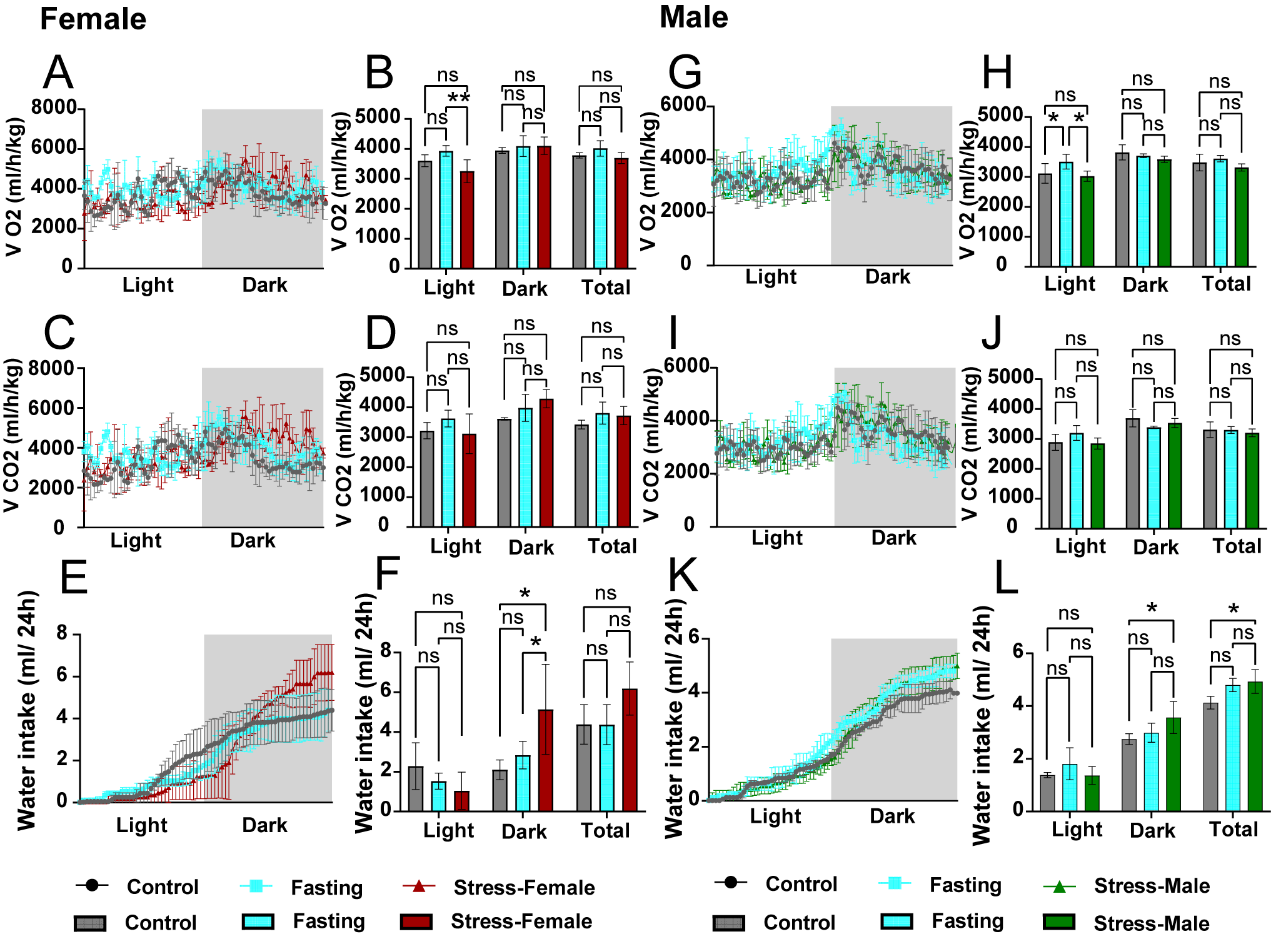


**Supplementary Figure 3. During the recovery period after chronic restraint stress, there is no significant energy expenditure.**

Oxygen consumption (VO_2_) and carbon dioxide expiration (VCO_2_) were determined by metabolic cages in females (**A-D**) and males (**G-J**), and the water intake during 24 hours after chronic restraint stress for females (**E-F**) and males (**K-L**). Data are expressed as the mean ± SEM (*n*=3-6) and were determined by one-way ANOVA or two-way ANOVA with Bonferroni post hoc tests. **P*<0.05, ***P*<0.01, ****P*<0.001 and *****P*<0.0001 for stress mice versus WT control or fasting control mice. Ns, means not signiﬁcant. Error bars represent SEM.


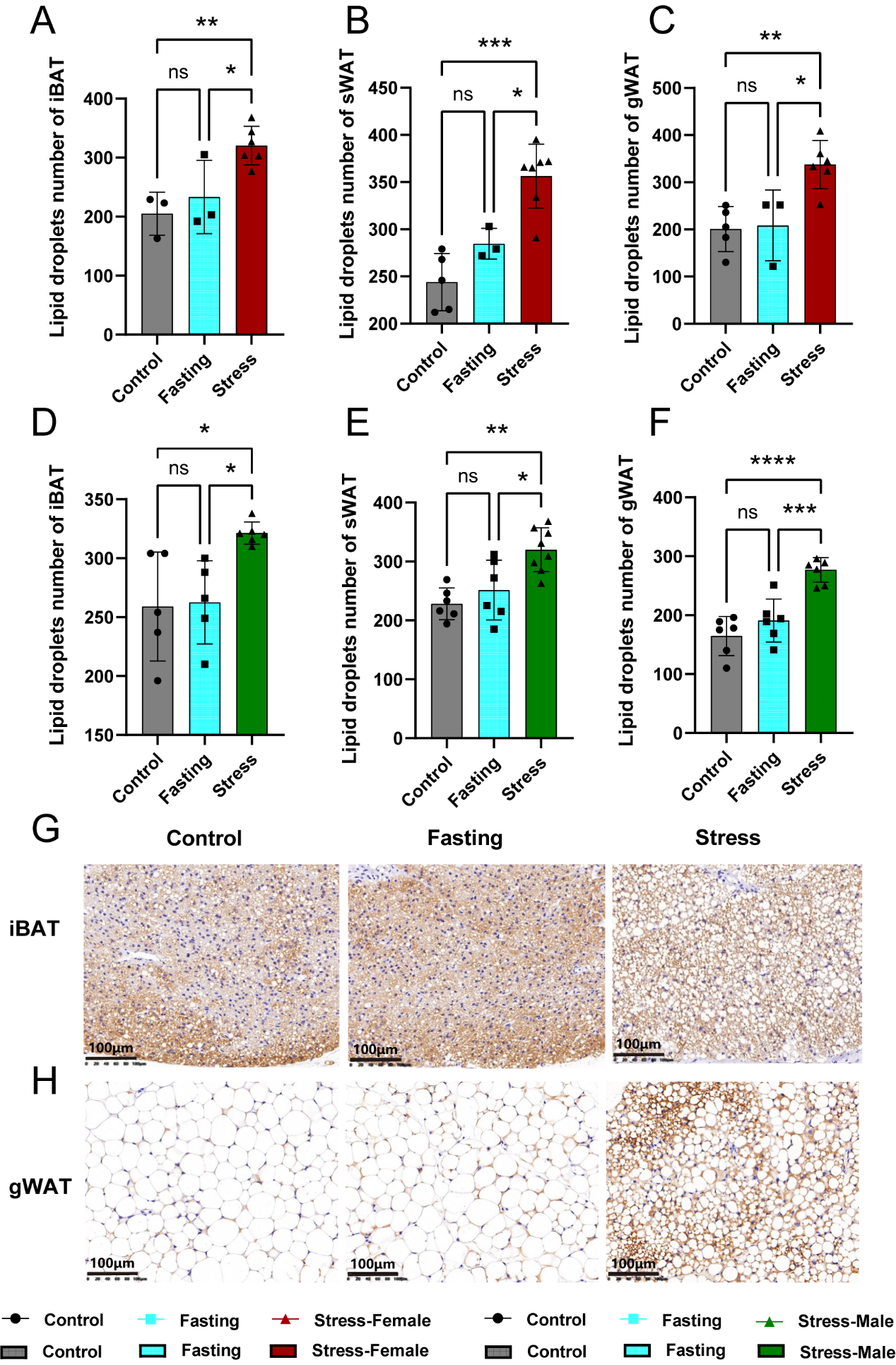


**Supplementary Figure 4. The protein expression of UCP1 in brown adipose tissue decreased, but increased in white adipose, and the number of adipocytes changed significantly.**

Lipid droplets number of H&E‐staining images under 200x magnification from iBAT, sWAT, and gWAT in females (**A-C**) and males (**D-F**). Representative immunohistochemistry images of iBAT and gWAT with 200x magnification from control, fasting and stress mice in females (**G-H**). Data are expressed as the mean ± SEM (*n*=3-6) and were determined by one-way ANOVA with Bonferroni post hoc tests. **P*<0.05, ***P*<0.01, ****P*<0.001 and *****P*<0.0001 for stress mice versus WT control or fasting control mice. Ns, means not signiﬁcant. Error bars represent SEM.


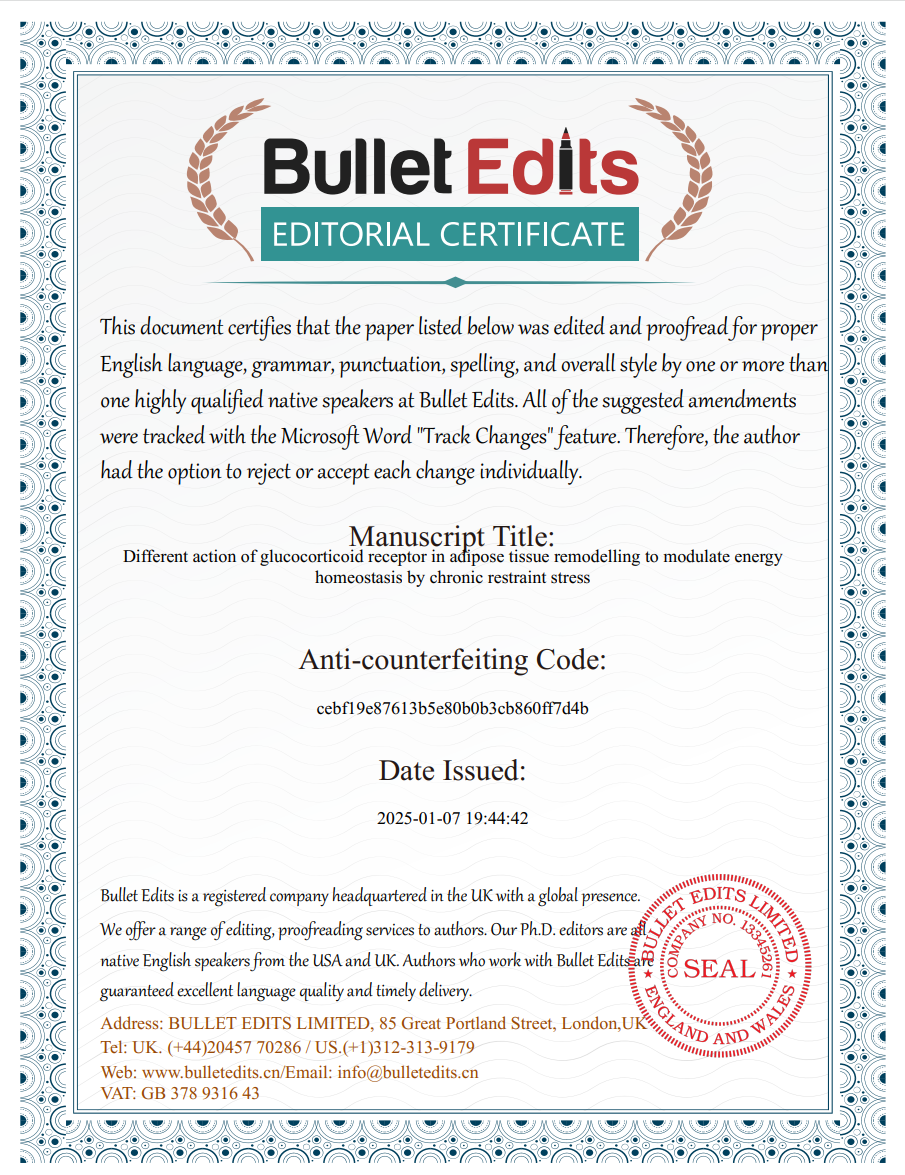


**Supplementary Figure 5. A certificate of language editing.**

**Supplementary Figure 6. All the raw data for uncorrupted western blotting.**

Overview of whole membrane example:

**
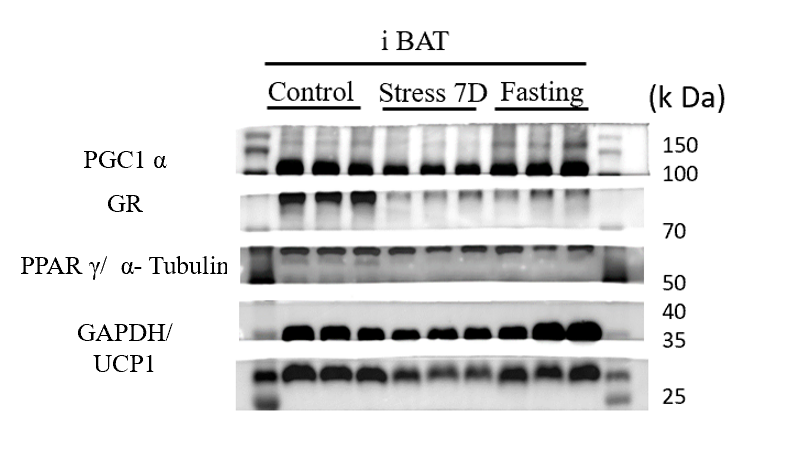
**

Fig S6-1. Figure 6A-Female-iBAT-GR





Fig S6-2.Figure 6A-Female-iBAT-PGC1α





Fig S6-3.Figure 6A-Female-iBAT-PPARγ





Fig S6-4. Figure 6A-Female-iBAT-UCP1





Fig S6-5.Figure 6A-Female-iBAT-αTublin





Fig S6-6. Figure 6C-Female-sWAT-GR





Fig S6-7.Figure 6C-Female-sWAT-PGC1α





Fig S6-8.Figure 6C-Female-sWAT-PPARγ





Fig S6-9. Figure 6C-Female-sWAT-UCP1





Fig S6-10.Figure 6C-Female-sWAT-αTublin





Fig S6-11. Figure 6E-Female-gWAT-GR





Fig S6-12.Figure 6E-Female-gWAT-PGC1α





Fig S6-13.Figure 6E-Female-gWAT-PPARγ





Fig S6-14. Figure 6E-Female-gWAT-UCP1





Fig S6-15.Figure 6E-Female-gWAT-αTubulin





Fig S6-16. Figure 6G-Male-iBAT-GR





Fig S6-17.Figure 6G-Male-iBAT-PGC1α





Fig S6-18.Figure 6G-Male-iBAT-PPARγ





Fig S6-19. Figure 6G-Male-iBAT-UCP1





Fig S6-20.Figure 6G-Male-iBAT-αTubulin





Fig S6-21. Figure 6I-Male-sWAT-GR





Fig S6-22.Figure 6I-Male-sWAT-PGC1α





Fig S6-23.Figure 6I-Male-sWAT-PPARγ





Fig S6-24. Figure 6I-Male-sWAT-UCP1





Fig S6-25.Figure 6I-Male-sWAT-αTubulin





Fig S6-26. Figure 6K-Male-gWAT-GR





Fig S6-27.Figure 6K-Male-gWAT-PGC1α





Fig S6-28.Figure 6K-Male-gWAT-PPARγ





Fig S6-29. Figure 6K-Male-gWAT-UCP1





Fig S6-30.Figure 6K-Male-gWAT-αTubulin
