# Supplementary material for: Different action of glucocorticoid receptor in adipose tissue remodelling to modulate energy homeostasis by chronic restraint stress
Source: Lipids Health Dis. 2025 Mar 27;24:121. doi: 10.1186/s12944-025-02539-0 (PMC11948944; doi:10.1186/s12944-025-02539-0)
Supplement: Supplementary file 2 — Supplementary Material 2: The proof report of the overall similarity index of the MS on iThenticate. [file 12944_2025_2539_MOESM2_ESM.pdf]

iThenticate®

864648025467781120.docx

Olivarez College

Document Details

Submission ID

trn:oid:::18876:79089344

Submission Date

Jan 13, 2025, 4:20 PM GMT+8

Download Date

Jan 13, 2025, 4:22 PM GMT+8

File Name

864648025467781120.docx

File Size

102.9 KB

18 Pages

4,383 Words

26,861 Characters

19% Overall Similarity

The combined total of all matches, including overlapping sources, for each database.

Filtered from the Report

- Bibliography
- Quoted Text

Match Groups

62 Not Cited or Quoted 16%
Matches with neither in-text citation nor quotation marks

12 Missing Quotations 3%
Matches that are still very similar to source material

0 Missing Citation 0%
Matches that have quotation marks, but no in-text citation

0 Cited and Quoted 0%
Matches with in-text citation present, but no quotation marks

Top Sources

14% Internet sources
13% Publications
8% Submitted works (Student Papers)

Integrity Flags

1 Integrity Flag for Review

Replaced Characters
24 suspect characters on 6 pages
Letters are swapped with similar characters from another alphabet.

Our system's algorithms look deeply at a document for any inconsistencies that would set it apart from a normal submission. If we notice something strange, we flag it for you to review.

A Flag is not necessarily an indicator of a problem. However, we'd recommend you focus your attention there for further review.

Match Groups

- **62** Not Cited or Quoted 16%
  Matches with neither in-text citation nor quotation marks
- **12** Missing Quotations 3%
  Matches that are still very similar to source material
- **0** Missing Citation 0%
  Matches that have quotation marks, but no in-text citation
- **0** Cited and Quoted 0%
  Matches with in-text citation present, but no quotation marks

Top Sources

14% Internet sources
13% Publications
8% Submitted works (Student Papers)

Top Sources

The sources with the highest number of matches within the submission. Overlapping sources will not be displayed.

1 Publication
Lele Ji, Ya Zhao, Linjie He, Jing Zhao et al. " Deficiency Attenuates Diet-Induced O... 3%

2 Internet
www.frontiersin.org 2%

3 Internet
jeccr.biomedcentral.com 2%

4 Internet
www.nature.com <1%

5 Internet
www.mdpi.com <1%

6 Publication
Sushil Sharma. "Charnolophagy in Health and Disease - With Special Reference to ... <1%

7 Internet
www.tandfonline.com <1%

8 Internet
worldwidescience.org <1%

9 Internet
docplayer.net <1%

10 Internet
www.cell.com <1%

11 Internet
www.researchsquare.com <1%

12 Internet
mdpi-res.com <1%

13 Internet
bdpsjournal.org <1%

14 Internet
downloads.hindawi.com <1%

15 Internet
pubmed.ncbi.nlm.nih.gov <1%

16 Internet
www.collectionscanada.gc.ca <1%

17 Internet
animalnutrition.imedpub.com <1%

18 Internet
scholar.sun.ac.za <1%

19 Publication
Hongli Sun, Ning Jia, Lixia Guan, Qing Su, Dan Wang, Hui Li, Zhongliang Zhu. "Inv... <1%

20 Submitted works
University of Malaya on 2015-11-13 <1%

21 Internet
respiratory-research.biomedcentral.com <1%

22 Internet
spandidos-publications.com <1%

23 Internet
www.magonlinelibrary.com <1%

24 Submitted works
Wright State University on 2013-12-11 <1%

25	Internet	
coek.info		<1%
26	Internet	
sci-hub.se		<1%
27	Internet	
www.science.org		<1%
28	Submitted works	
RMIT University on 2017-04-11		<1%
29	Publication	
Carolyn D. Berdanier. "Mitochondria in Health and Disease", CRC Press, 2019		<1%
30	Submitted works	
Egerton University on 2023-12-14		<1%
31	Submitted works	
University of Bath on 2014-10-28		<1%
32	Internet	
elifesciences.org		<1%
33	Internet	
insight.jci.org		<1%
34	Publication	
Chen Sun, Jiaqi Liang, Jia Zheng, Shuyu Mao, Siyu Chen, Ainiwaer Aikemu, Chang ...		<1%
35	Publication	
Ming Zhao, Xiaoli Chen. "Eicosapentaenoic acid promotes thermogenic and fatty ...		<1%
36	Publication	
Minghui Cao, Roi Isaac, Wei Yan, Xianhui Ruan et al. "Cancer-cell-secreted extrace...		<1%
37	Publication	
Yang Xiao, Dongmin Liu, Mark A. Cline, Elizabeth R. Gilbert. "Chronic stress and a...		<1%
38	Publication	
Nutrigenomics, 2016.		<1%

39 Submitted works

University of Hong Kong on 2012-10-30 <1%

Different action of glucocorticoid receptor in adipose tissue remodelling to modulate energy homeostasis by chronic restraint stress

Abstract

Background: Chronic stress in daily life is a well-known trigger for various health issues. Despite advancements in obesity research, the mechanisms governing lipid metabolism in adipose tissue during cachexia remain poorly understood.

Methods: A chronic restraint stress (CRS) model was used to induce significant physiological and psychological stress in mice. Mice were subjected to 6 hours of restraint daily in 50 mL plastic tubes for seven consecutive days. A fasting control group was included for comparison. Post-stress assessments included behavioural tests, glucose and insulin tolerance tests and indirect calorimetry. Blood and adipose tissue samples were collected for mRNA and protein analyses.

Results: CRS induced significant psychological and physiological changes in mice, including depression-like behaviours, weight loss and reduced insulin sensitivity. Notably, CRS caused extensive adipose tissue remodelling. White adipose tissue (WAT) underwent significant browning, counteracting the stress-induced whitening of brown adipose tissue (BAT), which exhibited impaired thermogenesis and functionality. The glucocorticoid receptor (GR) plays a crucial role in lipid metabolism regulation during these changes. GR expression levels were inversely correlated in BAT and WAT and aligned with thermogenic gene expression patterns across adipose tissues. These findings suggest that under chronic metabolic stress, GR mediates tissue-specific responses in adipose tissues, driving functional and phenotypic transitions in BAT and WAT to maintain energy homeostasis.

Conclusions: This study provides novel insights into the contrasting thermogenic phenotypes of BAT and WAT under emaciation and highlights the critical role of GR in adipose tissue remodelling during CRS and its potential as a therapeutic target. Addressing GR-mediated changes in adipose tissues may help alleviate BAT dysfunction in cachexia and promote WAT browning, enhancing metabolic stress resistance.

Keywords: Glucocorticoid receptor, biological stress, adipose tissue, thermogenesis

1. Introduction

Modern society's increasing competition and mounting psychological and physiological pressures have led to the widespread prevalence of chronic stress, characterised by persistent feelings of pressure and being overwhelmed over extended periods[1]. Chronic stress triggers a cascade of cellular, physiological and behavioural effects, resulting in various health issues, including emotional disorders[2,3], binge eating[4,5] or anorexia[6,7], endocrine and metabolic disorders[8,9], immunologic dissonance[10–12] and even cancer development[13–15].

Adipose tissue is traditionally classified into two primary types: brown adipose tissue (BAT), responsible for non-shivering thermogenesis and white adipose tissue (WAT), the primary site of energy storage[16,17]. Moreover, a third type, beige adipocytes, originates from white adipocyte progenitors and can undergo browning in response to specific stimuli, acquiring characteristics of brown adipocytes[18]. Brown adipocytes have an abundance of multichambered lipid droplets and elevated mitochondrial content, indicative of their ability to enhance energy expenditure[19]. This is facilitated by the presence of uncoupling protein 1 (UCP1)[20], enabling the dissipation of the proton gradient across the mitochondrial inner

membrane, thereby decoupling respiration from ATP synthesis[21]. Adipose tissue functions not only as an energy reservoir but also as an active endocrine organ, regulating lipid metabolism, thermoregulation and glucose homeostasis[22]. While considerable research has focused on adipose tissue metabolism in obesity, limited attention has been given to its role during emaciation, a state characterised by reduced adipose mass and depot-specific alterations in adipocyte type and function.

Glucocorticoids (GCs), the primary stress hormones, are secreted in response to psychological and physiological stressors and mediate their effects through the glucocorticoid receptor (GR), a ligand-activated transcription factor. The GC-GR signalling network involves multiple genomic and non-genomic pathways influenced by exposure duration, adipose tissue location and species-specific factors [23]. GR's transcriptional activity exhibits strong tissue specificity due to its dependence on environmental determinants. The hypothalamic-pituitary-adrenal (HPA) axis plays a critical role in chronic stress by regulating GC secretion through a cascade of hormonal events[24]. GCs influence adipogenesis and lipolysis, but their dual role remains contentious due to the complexity of these processes[25–28].

This study investigates the physiological and metabolic alterations under chronic stress, using a chronic restraint stress (CRS) model[29,30] to induce significant physiological and psychological trauma in mice. Alongside behavioural changes and weight loss, CRS led to pronounced adipose tissue remodelling. Notably, BAT exhibited significant 'whitening' and thermogenic dysfunction, while WAT demonstrated adaptive browning. Diverging from previous fat-related research, this study provides novel insights into the contrasting thermogenic phenotypes of BAT and WAT under cachectic conditions and underscores the

pivotal role of GR in adipose tissue remodelling. The findings of this study reveal that targeting GR within remodelled adipose tissues may mitigate BAT dysfunction, enhance WAT browning and alleviate metabolic stress in patients experiencing severe metabolic stress.

2. Materials and methods

2.1. Mice care

C57BL/6J male and female mice (8 weeks old) were obtained from GemPharmatech (Jiangsu, China) and housed in groups of five in a specific pathogen-free facility. Mice were maintained under a 12-hour light-dark cycle at a controlled room temperature (22 ± 0.5 °C) with unrestricted access to water and a standard diet (10% calories from fat) unless otherwise stated. All animal experiments were conducted according to the guidelines of the Institutional Animal Care and Use Committee of the Centre for Experimental Research in Clinical Medicine, Shengli Clinical Medical College of Fujian Medical University, Fuzhou, China (Permission Number: IACUC-FPH-PZ-20240624【0012】).

2.2. Experimental design

The CRS model was established by restraining mice in 50 mL plastic centrifuge tubes with ventilation holes for 6 hours daily (8:00 a.m. to 2:00 p.m.) over seven consecutive days[29–31]. A fasting control group was included to account for the metabolic effects of food and water deprivation during the restraint period. Mice were randomly assigned to groups (15–18 mice per group, total n = 100). Following the restraint period, behavioural tests, glucose tolerance tests (GTT), insulin tolerance tests (ITT) and metabolic cage studies were performed in batches (Figure 1). After the experiments ended, mice were deeply anaesthetised with pentobarbital (Sigma Aldrich, Missouri, USA). Blood samples were collected for plasma hormone analysis

and brown and white adipose tissues, as well as liver samples, were harvested. Tissues were flash-frozen in liquid nitrogen and stored at −80 °C for subsequent analyses.

2.3. Behavioural experiments

Stress-exposed male and female mice underwent behavioural assessments, including the open field test (OFT), elevated plus maze (EPM) and tail suspension test (TST), to evaluate locomotor activity and depression-like behaviours. All tests were conducted in a dimly lit room, with mice acclimated overnight prior to testing. Experiments were performed between 8:00 a.m. and 6:00 p.m. to minimise circadian influences. After each test, the surfaces of the equipment were cleaned with 75% ethanol to eliminate odour trails.

2.3.1. OFT

Mice were individually placed at the centre of a square arena (50 × 50 × 50 cm) constructed from grey polyvinyl chloride and monitored for 5 minutes using an automated video tracking system. Locomotor activity was recorded, and the digitised movement data were analysed using the DigBehv animal behaviour analysis programme (Shanghai, China). The central area, defined as one-quarter of the arena's total size, was used to measure depression-like behaviour by tracking the time spent and the number of entries into the central zone.

2.3.2. EPM

The EPM consisted of two open arms (10 × 30 cm) and two enclosed arms (10 × 30 × 20 cm), positioned opposite each other, with a central platform measuring 10 × 10 cm and elevated 40 cm above the ground. Mice were placed individually at the centre of the maze, facing an open arm, and their activity was recorded for 5 minutes[32]. The time spent in the open arms and the number of open arm entries were quantified using the Smart v3.0 system (Panlab, Barcelona,

Spain) (Supplementary Figure 1A–D).

2.3.3. TST

For the TST, each mouse's tail was affixed to a hanging hook approximately 1 cm from the tip, suspending the mouse in an inverted position about 10 cm above the ground. Behaviour was recorded for 6 minutes, and the last 4 minutes of immobility time were analysed. The latency to the first instance of immobility was also calculated using the Smart v3.0 system (Panlab, Barcelona, Spain) to assess depression-like behaviour[33] (Supplementary Figure 1E-H).

2.4. Intraperitoneal glucose and insulin tolerance test

The GTT test was performed on mice of both sexes that were fasted for 12 hours as described previously[34,35]. Glucose at a dose of 2 g/kg of body weight was administered intraperitoneal injection. For ITT, mice were fasted for 6 hours (8:00-14:00) and received an intraperitoneal injection of insulin (Novo Nordisk, Copenhagen, Denmark) of 1U/kg of body weight. Blood glucose concentrations were determined at 15, 30, 60, 90, 120, and 180-minute time pointes after glucose or insulin injection using a glucometer from Roche (Basel, Switzerland) and calculated the area under the curve (AUC).

2.5. Indirect calorimetry test

The metabolic parameters of mice were monitored using TSE PhenoMaster animal monitoring system (Hofheim, Germany). Mice were acclimated for 24 hours before measurements were taken. The oxygen consumption (VO2), exhaled carbon dioxide (VCO2), respiratory exchange ratio (RER)[36] and energy expenditure (EE)[37]of each mouse were determined for a 24-hour period. The RER and EE were calculated based on the VO2 and VCO2 data. Analysis of covariance (ANCOVA) was used to compare metabolic parameters of mice, in which body

iThenticate Page 12 of 24 - Integrity Submission Submission ID trn:oid:::18876:79089344

weight was used as covariate.

2.6. Biochemical analyses of the plasma

The blood of mice in each group was collected from the medial canthus vein with stand for 4 hours, then centrifuged at 3000 rpm for 15 minutes at 4 ℃, and taken out the supernatant. The serum level of corticosterone (CORT), luteinizing hormone (LH), follicle stimulating hormone (FSH) detected respectively by enzyme-linked immunosorbent assay (ELISA) kits from Meimian (Jiangsu, China) and testosterone (T), oestradiol (E2) detected by ELISA kit from Beyotime (Shanghai, China), listed in supplementary Table 1. The procedures were performed according to the manufacturer's instructions.

2.7. Hematoxylin and eosin staining (H&E) of adipose tissues

Adipose tissues were fixed in 4% paraformaldehyde for 24 hours, embedded in paraffin and sectioned at a thickness of 5 μm. Following deparaffinisation and rehydration, slides were stained with H&E and imaged using a Nikon Eclipse Ci-L microscope (Tokyo, Japan). Adipocyte counts and lipid droplet areas were quantified using AdipoCount software (Shanghai, China) in at least three fields per slide at 200× magnification [38]

2.8. Quantitative real-time polymerase chain reaction (RT-PCR)

Samples were extracted using RNA isolater Total RNA Extraction Reagent (Vazyme, Jiangsu, China). Total RNA (1000 ng) was reverse transcribed to generate complementary DNA using PrimeScipt RT Reagent Kit with genomic DNA Eraser (Takara, Kusatsu, Japan). Quantitative RT-PCR was performed on CFX 96 Real-Time system (Bio-Rad, California, USA) using SYBR Premix Ex Taq II Kit (Takara, Kusatsu, Japan) with specific primers synthesized by Shangya (Fuzhou, China), listed in Supplementary Table 2. *β* actin was used as an internal

control for adipose tissues.

2.9. Immunohistochemistry and immunofluorescence

For immunohistochemistry, adipose tissues were fixed with 4% paraformaldehyde overnight, permeabilized with 0.2% Triton X-100 for 10 min, and blocked with 5% normal goat serum at room temperature for 60 minutes. After that, samples were incubated with anti-UCP1 (1:100, Cell Signaling Technology (CST), Massachusetts, USA) antibody at 4 °C overnight. The following day, images were collected following incubation with the secondary antibody and staining with diaminobenzidine (DAB) and hematoxylin (Supplementary Figure 4A-B). For immunofluorescence, paraffin embedded tissue slides were deparaffinised and subjected to antigen retrieval in citric acid buffer, blocked with 5% normal goat serum. Incubated with anti-GR (1:50, Santa Cruz, California, USA) or anti-UCP1 (1:100, CST, Massachusetts, USA) antibody at 4 °C overnight and then incubated with the corresponding secondary antibody at room temperature for 1 hour, finally stained with diamidino-2-phenylindole (DAPI) 20 minutes. Images were captured using a Nikon Eclipse Ci-L microscope (Tokyo, Japan). The used primary antibodies were listed in Supplementary Table 3.

2.10. Western blot (WB) analysis

Total protein was extracted and protein concentrations were measured using protein assay kits from Solarbio (Beijing, China). The following procedures were performed: total protein was separated and transferred to PVDF membranes (Millipore, Massachusetts, USA). After blocking the membrane with 5% bovine serum albumin (BSA, Beyotime, Shanghai, China), incubated the membrane with various primary antibodies (Supplementary Table 3) at 4 °C overnight and then incubated with secondary antibodies at room temperature for 30 minutes.

Finally, protein detection was performed using a chemiluminescence instrument.

2.11. Statistical analysis

Prior to analysis, data were subjected to normality tests. Differences between stressed mice and wild-type (WT) controls or fasting controls were evaluated using Student's *t*-test, ordinary one-way ANOVA, or two-way ANOVA with Bonferroni post hoc tests, performed in GraphPad Prism 9 (GraphPad Software, California, USA). Data are presented as mean ± standard error of the mean (SEM), and $P < 0.05$ was considered statistically significant.

3. Results

3.1. Fasting exerts a subtle influence on metabolic shifts in mice under CRS

CRS significantly affected mice, leading to alterations in emotional behaviour, body weight and adipose tissue remodelling. Both female and male mice exhibited consistent outcomes. Notably, the phenotypes of the fasting control group were largely aligned with those of the blank control group, while both groups displayed pronounced metabolic differences compared to the restraint stress group. These findings indicate that the phenotypic changes observed during the restraint stress period were not attributable to fasting or water deprivation.

3.2. Mice subjected to CRS exhibit depression-like behavioural manifestations

Behavioural and locomotor changes in CRS-exposed mice were assessed through the OFT (Figure 2), EPM and TST (Supplementary Figure 1). Stress-exposed mice demonstrated reduced track distance and average speed in the OFT (Figure 2A–B, H–I), decreased exploratory behaviour (Figure 2C, J) and a tendency to avoid the centre area (Figure 2D–G, K–N). EPM testing revealed a reduction in time spent in the open arms, while TST showed prolonged immobility time in female mice (Supplementary Figure 1A–H). Collectively, these

results indicate depression-like behavioural phenotypes in CRS-exposed mice.

3.3. CRS induced a lean phenotype and hormone disturbance in mice

CRS-exposed mice exhibited a negative energy balance. After seven days of restraint, total food intake (Supplementary Figure 2A–B) and body weight (Figure 3A–B, H–I) were significantly reduced, reflecting heightened energy expenditure during the restraint period. Organ weights measured at the end of the experiment revealed contrasting phenotypes among adipose tissues: increased interscapular brown adipose tissue (iBAT) weight (Figure 3D, K) and reduced weights of subcutaneous (sWAT), gonadal (gWAT) and retroperitoneal (rWAT) white adipose tissues (Figure 3E, L). Moreover, serum corticosterone levels were elevated in female mice (Figure 3F) but not in males (Figure 3M). Testosterone levels significantly increased in both female and male mice (Figure 3G, N). In male mice, LH and FSH levels decreased (Supplementary Figure 2F–G), whereas no significant changes were observed in female mice (Supplementary Figure 2C–E), possibly due to differences related to the oestrous cycle and sex-specific responses.

3.4. CRS impairs insulin sensitivity and enhances RER, with carbon-based foods as the primary energy source

In CRS-exposed mice, glucose tolerance remained largely unchanged, except for a higher glucose level at the 30-minute time point compared to the control group (Figure 4A, H–I). Female fasting mice exhibited lower blood glucose levels (Figure 4B). However, insulin sensitivity was markedly impaired in stress-exposed mice of both sexes, as evidenced by reduced glucose excursions (Figure 4C, K) and a significantly lower inverse AUC compared to controls (Figure 4D, L).

Metabolic cage analyses revealed no significant changes in oxygen consumption (Supplementary Figure 3A–B, G–H) or carbon dioxide production (Supplementary Figure 3C-D, I–J) following seven days of CRS. Consequently, total energy expenditure remained unchanged (Figure 4G–H, O–P). However, the RER significantly increased, particularly during the dark cycle (Figure 4E–F, M–N). There was also a trend toward increased food and water intake over 24 hours, although these changes were not statistically significant (Supplementary Figure 3E–F, K–L). These findings suggest that CRS-exposed mice predominantly relied on carbohydrates as their primary energy source to compensate for the reduction in WAT caused by excessive energy expenditure during the stress period[37,39]. In contrast, fasting mice exhibited lower RER values [40] and male fasting mice (Figure 4M-P) demonstrated slightly higher energy expenditure [41,42].

3.5. Mice exposed to CRS underwent significant adipose tissue remodelling as a metabolic stress adaptation

Representative H&E staining of iBAT, sWAT and gWAT revealed significant changes in adipose tissue morphology in CRS-exposed mice (Figure 5A, F). Adipose tissue mass is influenced by both the average size and the number of constituent adipocytes[43]. A notable browning effect was observed in WAT, characterised by a reduction in lipid droplet size (Figure 5B, G) and an increase in the number of lipid droplets (Supplementary Figure 4D–E, G–H). In contrast, BAT exhibited an accumulation of lipid droplets (Figure 5B, G; Supplementary Figure 4C, F), consistent with changes in tissue weight (Figure 3C–E, J–L). Moreover, the expression of thermogenic genes, including *Ucp1*, *Peroxisome proliferator-activated receptor gamma coactivator 1-alpha* (*Pgc1α*), *Peroxisome proliferator-activated receptor gamma* (*Pparγ*) and

39 *Cytochrome c oxidase subunit 8b* (*Cox8b*), was significantly upregulated in WAT, particularly in gWAT (Figure 5D–E, I–J). Conversely, these genes were downregulated in BAT (Figure 5C, H). Immunohistological analysis of UCP1 protein in BAT and gWAT tissues mirrored the trends observed in mRNA expression (Supplementary Figure 4A, B).

3.6. Western blot analysis uncovers GR correlation with thermogenic proteins, indicating its role in adipose tissue remodelling

6 In chronic stress, the HPA axis is activated [44], and GRs play an important role in adipose tissue lipid metabolism [23,45]. Therefore, this study examined GR protein expression in various adipose tissues and found it aligned with thermogenic protein expression in both BAT and WAT. Specifically, in the 'whitening' BAT of metabolically stressed mice, the expression of UCP1, PGC1α and PPARγ proteins was reduced, accompanied by a significant decrease in GR expression (Figure 6A–B, G–H). Conversely, in the 'browning' sWAT and gWAT, UCP1, PGC1α and PPARγ protein levels were elevated, correlating with increased GR expression (Figure 6C-E, I-L). Furthermore, immunofluorescence colocalisation assays further demonstrated that depolarised BAT in stressed mice exhibited a significant reduction in cytoplasmic GRs (Figure 7A-B).

3.7. Downregulation of UCP1 and GR in BAT during 'whitening' correlates with mitochondrial dysfunction, autophagy reduction and inflammatory exacerbation

6 To explore the mechanisms underlying the 'whitening' phenotype of BAT in stressed mice, the study identified mitochondrial dysfunction, evidenced by reduced mRNA expression of key 25 mitochondrial dynamics regulators, including *Dynamin-related protein 1* (*Drp1*), *Optic atrophy 1* (*Opa1*) and *Mitofusin 1* (*Mfn1*) (Figure 7C, F). Autophagy was also impaired, as

indicated by decreased mRNA expression of autophagy-related genes, such as *Microtubule-associated protein light chain 3* (*LC3*), *Sequestosome 1* (*p62*), *Autophagy-related* *protein 5* (*Atg5*) and *Autophagy-related protein 7* (*Atg7*) (Figure 7D, G). Additionally, inflammation was exacerbated, with increased levels of interleukin-1β (IL-1β) observed in female mice (Figure 7E). These alterations may be linked to the reduced expression of GR, which could further aggravate the dysfunctional phenotype of BAT under chronic stress conditions.

4. Discussion

Adipose tissue remodelling is influenced by various exogenous and endogenous factors, including energy status fluctuations and hormonal changes[22] and is closely associated with the pathophysiology of metabolic disorders. Once regarded merely as a passive lipid reservoir, adipose tissue is now recognised as a metabolically active organ playing a central role in whole-body energy homeostasis. It contributes to critical processes such as immune responses, glucose metabolism, insulin sensitivity and thermogenesis[19]. While lipolysis is known to fulfil energy demands during negative energy balance[7], the mechanisms underlying adipose tissue remodelling in states of emaciation remain poorly understood.

Recent studies highlight the heterogeneity of adipose tissue, emphasising its regional variations in metabolic processes and hormonal responses [22]. This emerging perspective has drawn significant attention. The present study primarily investigated the pathophysiological implications of adipose tissue metabolism and associated mechanisms under CRS.

BAT, specialised for energy expenditure, is pivotal in maintaining energy homeostasis through adaptive thermogenesis[18]. This study demonstrated that mice subjected to CRS exhibited a pronounced 'whitening' phenotype in BAT, coupled with impaired thermogenic function. This

may be attributed to the intense physical struggle during restraint, which likely triggered a substantial increase in skeletal muscle shivering thermogenesis. Consequently, non-shivering thermogenesis in BAT was reduced, thereby decreasing BAT's energy consumption for thermogenesis. These findings align with prior research, suggesting that individuals with anorexia nervosa experience a compensatory reduction in basal metabolic rate to mitigate chronic energy deficits caused by restrictive eating behaviours[46,47]. Furthermore, the study revealed that chronic and excessive GC exposure contributes to lipid accumulation in specific body regions, particularly in BAT. This GC-induced lipid deposition promotes a 'whitening' phenotype in BAT, impairing its thermogenic capability[48,49].

WAT plays a crucial role in regulating various physiological processes that impact energy balance. It is also a significant target for other organs, contributing to energy homeostasis by managing the storage and utilisation of fatty acids [17]. This study demonstrated that under conditions of chronic stress and substantial energy expenditure, the mobilisation of glucocorticoid hormones for adipose remodelling in mice serves a protective role against metabolic stress. Both sWAT and gWAT participated in the browning process, compensating for BAT dysfunction to maintain energy homeostasis. These findings align with the research of Schulz et al.[50] and Wu et al.[51]. Furthermore, a notable reduction in fat mass among stressed mice led to an increase in the RER, with carbohydrates becoming the primary energy substrate. Conversely, fasting male mice exhibited a decrease in RER due to restricted dietary intake. As a pivotal endocrine tissue, WAT secretes various hormones and inflammatory factors and expresses multiple receptors for insulin, leptin, steroid hormones (e.g., glucocorticoids, androgens, estrogens) and catecholamines [52], enabling homeostatic regulation.

Chronic stress is widely recognised to activate the HPA axis[44], leading to GC release, which reallocates energy reserves to meet immediate or anticipated demands. This study highlights the interaction between GRs and adipose remodelling, suggesting that GRs specific-changes related to inducing insulin resistance, regulating lipids metabolism, and exacerbating inflammation. In general, chronic stress results in heightened HPA axis activity and diminished hypothalamic-pituitary-gonadal (HPG) axis activity. In this stress model, elevated serum GC levels were observed in female mice, whereas no significant differences were detected in males. This discrepancy may be attributed to circadian fluctuations of the HPA axis and inconsistent blood collection times. Regarding reproductive hormones, both sexes exhibited significantly increased testosterone levels. However, other reproductive hormones were reduced in males but not in females, potentially due to the hormonal fluctuations characteristic of the female oestrous cycle. Hormones significantly influence body fat[53]. This study revealed increased levels of glucocorticoids and testosterone, both of which are known lipolytic hormones[54,55]. Although GRs are nearly ubiquitous throughout the body, GCs exert cell- and tissue-specific effects[56]. Moreover, stress models can result in distinct adipose phenotypes. For instance, Rebuffé-Scrive M et al. observed an increase in mesenteric fat pad mass without changes in the epididymal, retroperitoneal, or inguinal regions in their stress model[57], contrasting with findings in the current model. Testosterone, a fat-reducing hormone, appears to have a more pronounced effect on visceral adipose tissue[55].

The classic and well-established mechanism for enhancing thermogenesis in organisms involves the activation of β3-adrenergic receptors (AR) by sympathetic nerve signals. This activation leads to an upregulation of PGC1α-driven mitochondrial biogenesis, while UCP1

facilitates the decoupling of the electron transport chain. This process generates heat and increases energy expenditure, further amplified by proton conductance using long-chain fatty acids derived from lipolysis[19]. Individuals exposed to chronic stress often experience dysregulation of the HPA axis, accompanied by heightened secretion of glucocorticoids and catecholamines. GCs enhance the lipolytic response in WAT to various hormones. Previous research has demonstrated that GRs are essential for signal transduction from β-AR to adenylate cyclase, leading to the activation of lipolysis [58]. These findings align with the diminished ability of GR-lacking adipocytes to undergo lipolysis under postprandial and fasting states, as a consequence of disrupted signalling from β-AR to adenylate cyclase, as elucidated by Mueller et al. [44]. In this study, the alterations in GR expression within adipose tissue were consistent with the expression of thermogenic genes, suggesting that GR may play a key role in mediating fat remodelling in functional adipose tissue.

The process of BAT ‘whitening’ has been associated with both autophagy[59] and mitochondrial dysfunction[60]. To further explore the potential mechanisms underlying the dysfunction of ‘whitening’ BAT in this stress model, the mRNA expression of genes related to mitochondria and autophagy was analysed. The results revealed mitochondrial dysfunction in ‘whitening’ BAT, accompanied by impaired autophagy. This impairment likely prevents the clearance of dysfunctional mitochondria. Furthermore, compromised autophagic lipolysis may contribute to excessive lipid accumulation in BAT[61], ultimately leading to impaired thermogenesis. Additionally, the reduction in GR expression may trigger an increase in inflammatory factor expression, potentially exacerbating this dysfunction.

Study strengths and limitations

This study provides a comprehensive evaluation of the physiological and metabolic alterations induced by CRS in mice. These include changes in emotional behaviour, body composition, glucose metabolism, metabolic state and, most notably, the functional remodelling of different adipose tissues along with their underlying mechanisms. The findings underscore the critical yet distinct roles of GRs in adipocytes in maintaining lipid metabolic homeostasis, depending on the specific type of adipose tissue and its energetic state under CRS. The most prominent effects are summarised in Figure 8.

Despite its strengths, this study has several limitations. While it provides valuable preliminary insights into the mechanisms driving BAT dysfunction, it does not delve deeply into the processes underlying the ‘browning’ of WAT. Considering the pivotal role of adipose tissue as a potent endocrine organ, future research should focus on identifying the secretory factors that mediate direct interactions between BAT undergoing ‘whitening’ and WAT undergoing ‘browning’ and further characterizing the molecular mechanisms underlying GR-mediated adipose tissue remodelling including the specific signalling pathways and downstream effectors involved in the ‘whitening’ and ‘browning’ processes.

5. Conclusions

This study highlights the significant impact of CRS on adipose tissue remodelling in mice, characterised by a pronounced ‘whitening’ phenotype in BAT, with impaired thermogenic function and a significant ‘browning’ response in various WAT depots. These findings suggest that the WAT browning response may serve as a protective adaptation to metabolic stress. The 6 GR within adipocytes plays a pivotal role in this process by regulating systemic fuel partitioning and energy metabolism. The insights gained from this research not only deepen

our understanding of the metabolic disruptions caused by chronic psychological and physiological stress but also identify potential therapeutic avenues. Targeting GR activity within remodelled adipose tissues could mitigate BAT dysfunction in cachectic patients and enhance WAT browning, thereby improving the body's ability to withstand metabolic stress and maintain energy homeostasis.
